# The role of lysine palmitoylation/myristoylation in the function of the TEAD transcription factors

**DOI:** 10.1038/s41598-022-09127-7

**Published:** 2022-03-23

**Authors:** Yannick Mesrouze, Gustavo Aguilar, Marco Meyerhofer, Fedir Bokhovchuk, Catherine Zimmermann, Patrizia Fontana, Alexandra Vissières, Hans Voshol, Dirk Erdmann, Markus Affolter, Patrick Chène

**Affiliations:** 1 Disease Area Oncology, Novartis Institutes for Biomedical Research, Novartis, 4002 Basel, Switzerland; 2 Biozentrum, University of Basel, Spitalstrasse 41, 4056 Basel, Switzerland; 3 Analytical Sciences and Imaging, Novartis Institutes for Biomedical Research, Novartis, WSJ 386 4.02.01, 4002 Basel, Switzerland

**Keywords:** Biochemistry, Biophysics

## Abstract

The TEAD transcription factors are the most downstream elements of the Hippo pathway. Their transcriptional activity is modulated by different regulator proteins and by the palmitoylation/myristoylation of a specific cysteine residue. In this report, we show that a conserved lysine present in these transcription factors can also be acylated, probably following the intramolecular transfer of the acyl moiety from the cysteine. Using Scalloped (Sd), the *Drosophila* homolog of human TEAD, as a model, we designed a mutant protein (Glu352Gln^Sd^) that is predominantly acylated on the lysine (Lys350^Sd^). This protein binds in vitro to the three Sd regulators—Yki, Vg and Tgi—with a similar affinity as the wild type Sd, but it has a significantly higher thermal stability than Sd acylated on the cysteine. This mutant was also introduced in the endogenous locus of the *sd* gene in *Drosophila* using CRISPR/Cas9. Homozygous mutants reach adulthood, do not present obvious morphological defects and the mutant protein has both the same level of expression and localization as wild type Sd. This reveals that this mutant protein is both functional and able to control cell growth in a similar fashion as wild type Sd. Therefore, enhancing the lysine acylation of Sd has no detrimental effect on the Hippo pathway. However, we did observe a slight but significant increase of wing size in flies homozygous for the mutant protein suggesting that a higher acylation of the lysine affects the activity of the Hippo pathway. Altogether, our findings indicate that TEAD/Sd can be acylated either on a cysteine or on a lysine, and suggest that these two different forms may have similar properties in cells.

## Introduction

**Figure 1:**
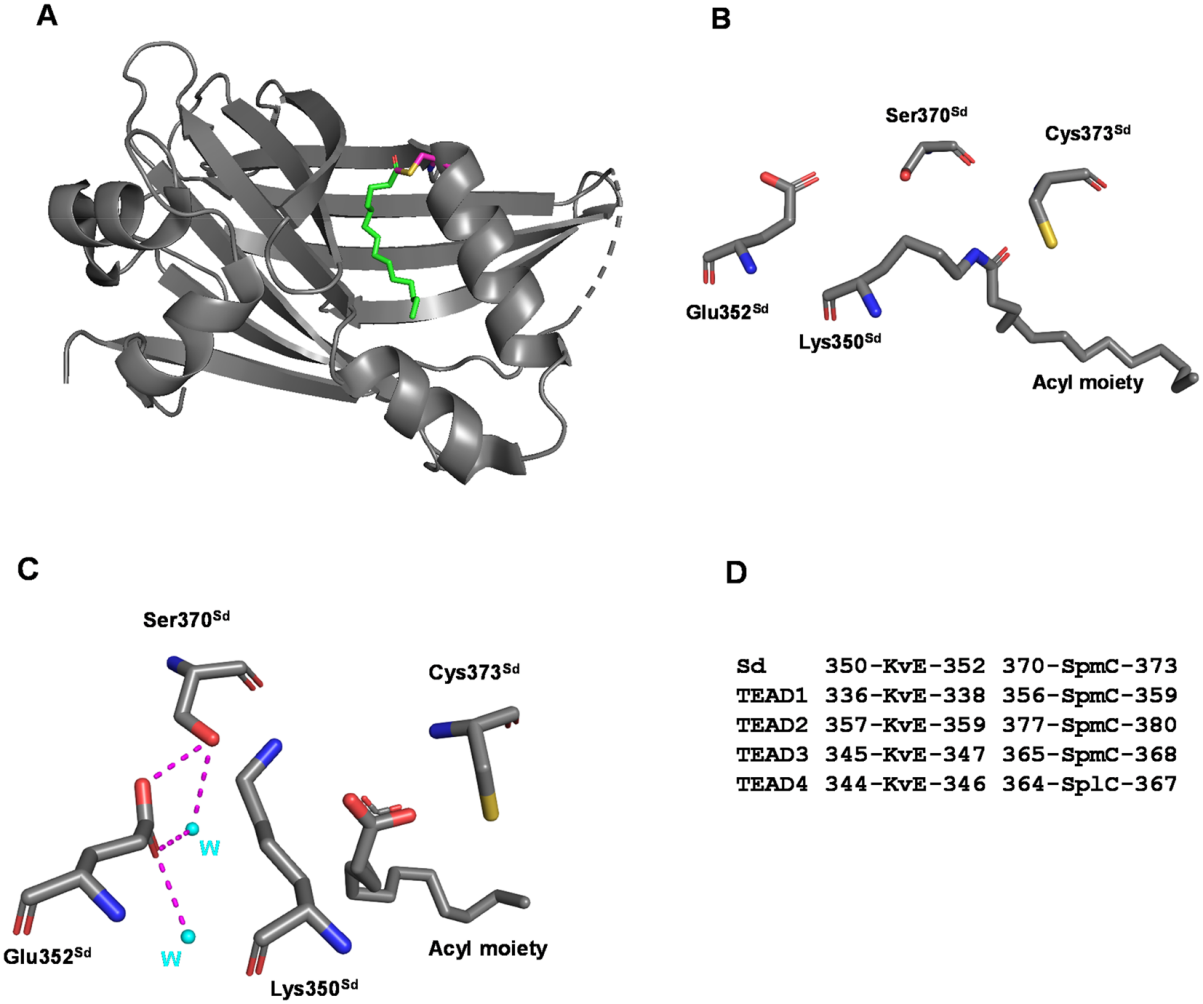
Structure of acylated TEAD/Sd. (**A**) Acylated TEAD4 (PDB 5oaq). TEAD4 is in grey and the acyl moiety in green. The acylated cysteine (Cys367^TEAD4^) is represented in magenta. (**B**) Acylation of Lys350^Sd^ (PDB 6y20 chain_A). The Sd residues located around Lys350^Sd^ are represented. Cys373^Sd^ is the other acylation site conserved in TEAD/Sd. (**C**) Acyl moiety non-covalently bound to Sd (PDB 6y20 chain_B). The Sd residues and the water molecules (W, cyan spheres) surrounding Lys350^Sd^ are represented. The dotted magenta lines represent putative hydrogen bonds. The figures were drawn with PyMOL (Schrödinger Inc., Cambridge, MA). (**D**) Alignment of the amino acid sequence of Sd and human TEAD1-4. The sequences have been manually aligned. The residues corresponding to Lys350^Sd^, Glu352^Sd^, Ser370^Sd^, and Cys373^Sd^ are indicated in upper cases. UniProtKB Seq. Id. Sd: P30052, TEAD1: P28347; TEAD2: Q15562; TEAD3: Q99594; TEAD4: Q15561.

The Hippo pathway is key in the control of organ morphogenesis in animals^1–3^. The most distal elements of this pathway are the TEAD transcription factors^4,5^. These proteins, which are unable to induce transcription on their own, need to interact with different partners such as YAP, TAZ, VGLL1-3 or VGLL4 to regulate the expression of various genes^6–9^. These regulators bind to an overlapping region at the surface of the TEAD protein, which indicates that the modulation of TEAD activity mandates a precise control of these interactions. Recently, it has been found that TEAD activity is also modulated by a very different mechanism^10,11^. These transcription factors are acylated (palmitoylated or myristoylated) on a cysteine residue which is conserved amongst the TEAD proteins from animals such as mammals or insects (e.g. *Drosophila melanogaster*)^10,11^. The covalently bound acyl moiety occupies a hydrophobic cavity protruding into the interior of the TEAD protein (Fig. 1A). The acylation enhances the stability of TEAD as observed in biochemical assays and in cells^11,12^. Acylation is also required when YAP/TAZ binds to TEAD, although it is dispensable for the interaction with VGLL4^10^. However, synthetic peptides mimicking YAP/TAZ have been reported to bind with a similar affinity to acylated and non-acylated TEAD^12^. The study of TEAD mutants that cannot be acylated shows that the acylation is required for muscle differentiation in vitro^10^. Furthermore, in vivo experiments conducted with an acylation deficient mutant of Scalloped (Sd, *Drosophila* homolog of human TEAD) reveal that this mutant has an impaired tissue overgrowth mediated by Yorkie (Yki, *Drosophila* homolog of human YAP)^10^. In vitro, recombinant TEAD can undergo an autoacylation reaction when incubated in the presence of Palmitoyl/Myristoyl-Coenzyme A^10,12^. In a cellular context, the acylation of TEAD is modulated by cell density and enzymes such as the Acyl Protein Thioesterase APT2^13^. Since non-acylated TEAD has a lower stability than acylated TEAD, the cleavage of the thioester bond by thioesterases may destabilize TEAD, triggering its degradation in cells.

Noland et al. have noticed that a conserved lysine adjacent to the acylated cysteine could lower its pK_a_ hence affecting its reactivity^11^. The mutation of this lysine induces a reduction in the cellular levels of TEAD suggesting that it may play a role in TEAD acylation^11^. Looking at the structures deposited at the Protein Data Bank (PDB, www.rcsb.org), we observed that human TEAD and *Drosophila* Sd can be acylated on this lysine (Lys344^TEAD4^ in PDB 6sen, 6gei and 6seo; Lys350^Sd^ in PDB 6y20) (Fig. 1B). As TEAD is not acylated when the cysteine is mutated^11,12^, it is likely that the acyl moiety is first added to the cysteine and then transferred to the lysine following an intramolecular reaction between the lysine and the thioester. Thioesterases (e.g., APT2) cannot cleave the amide bond made between the lysine and the acyl, suggesting that acyl-lysine TEAD should escape the regulation exerted by these enzymes. Therefore, acyl-lysine TEAD may have a different in vivo behavior than acyl-cysteine TEAD. Since human cells can express up to four TEADs, they are challenging models to study the effect of the acylation of the lysine because more than one of these proteins may have to be modified simultaneously to trigger a significant biological effect. In contrast, *D. melanogaster* is an attractive model organism to study the impact of the acylation of the lysine on the function of this transcription factor because it only expresses Sd. We report here in vitro and in vivo studies of the effect of the acylation of the conserved lysine Lys350^Sd^ on the properties and functions of Sd.

## Results

### Study of the acylation of wild type and mutant Sd proteins

Hydroxylamine reacts with thioesters^14^ and this chemical has been successfully used to deacylate TEAD^11,15,16^. Therefore, we treated recombinant wild type Sd (wt^Sd^) with hydroxylamine. Our results, obtained using preparations of wt^Sd^ purified from several—independent—expressions in *Escherichia coli*, show that only a fraction of this protein is sensitive to hydroxylamine (Fig. S1): on average, about half of wt^Sd^ is deacylated after hydroxylamine treatment (Table 1). Since a large fraction of this protein is insensitive to hydroxylamine, we hypothesized that it may correspond to wt^Sd^ acylated on Lys350^Sd^ as seen in the X-ray structure (Fig. 1B). To check this hypothesis, we engineered the Lys350Ala^Sd^ mutation to prevent any acylation at position-350. The treatment of this mutant protein with hydroxylamine leads to complete de-acylation (Fig. S1, Table 1). This indicates that in the absence of Lys350^Sd^, Sd is only acylated on Cys373^Sd^, the conserved acylation site present in the TEAD/Sd proteins^10–12^. These findings, together with the structural data, reveal that Sd can be acylated on Cys373^Sd^ or on Lys350^Sd^, and that hydroxylamine treatment can be used to differentiate between the two forms: acyl-Cys373^Sd^ is sensitive to hydroxylamine while acyl-Lys350^Sd^ is insensitive to it.

**Table 1:** Acylation status and thermal stability of wild type and mutant Sd proteins.

Proteins	HA-sensitive (%)	HA-insensitive (%)	T_m_ (^o^C)
wt^Sd^	47 ± 3	54 ± 3	58 ± 1
Lys350Ala^Sd^	100 ± 0	0 ± 0	52.0 ± 0.1
Glu352Gln^Sd^	10 ± 1	90 ± 1	52 ± 1–63.3 ± 0.3
Glu352Leu^Sd^	20 ± 1	81 ± 1	n.d

The fully acylated Sd proteins were treated with hydroxylamine (HA) and the reaction mixtures were analyzed by LC–MS. HA-sensitive and HA-insensitive correspond to the fraction of the protein that reacted or did not react with hydroxylamine, respectively. The melting temperature (T_m_) of the different proteins has been measured in a Fluorescence Thermal Shift Assay. n.d. not determined (a T_m_ could not be accurately determined for the two Sd forms present in the Glu352Leu^Sd^ preparations). Averages and standard errors are given (n ≥ 2).

Since the wt^Sd^ preparations contain only 50% acyl-Lys350^Sd^, it is not possible to accurately characterize the effect of the acylation of Lys350^Sd^ on the properties of Sd. Therefore, we looked for a strategy to enhance the level of Lys350^Sd^ acylation. As already mentioned, the acyl moiety is covalently bound to Lys350^Sd^ in the published structure (PDB 6y20 chain_A, Fig. 1B), but it is also non-covalently bound to Sd in the same structure (PDB 6y20 chain_B, Fig. 1C). We hypothesized that the position of Lys350^Sd^ in its non-covalent form should be closer to its conformation prior to reacting with the thioester formed by the acyl moiety and Cys373^Sd^. We therefore focused our analysis on this part of the structure. Lys350^Sd^ is located next to Glu352^Sd^ and these two residues are near Ser370^Sd^ and water molecules. Glu352^Sd^ and Ser370^Sd^ are conserved in the four human TEAD proteins (Fig. 1D). The presence of Glu352^Sd^ next to Lys350^Sd^ led us to hypothesize that these two residues could form a salt bridge that may decrease the ability of Lys350^Sd^ to react with the thioester. Therefore, preventing this interaction may enhance Lys350^Sd^ reactivity. To evaluate this possibility, we engineered two mutants. The Glu352Gln^Sd^ mutation prevents the formation of a salt bridge with Lys350^Sd^ while still permitting the glutamine at position-352 to form hydrogen bonds with its surrounding neighbors. The Glu352Leu^Sd^ mutation prevents the formation of both a salt bridge with Lys350^Sd^ and the formation of hydrogen bonds. These two mutations significantly reduce the sensitivity of Sd to hydroxylamine treatments, suggesting that they enhance the formation of acyl-Lys350^Sd^ (Fig. S1, Table 1). Since the Glu352Gln^Sd^ mutation leads to a higher Lys350^Sd^ acylation than the Glu352Leu^Sd^ mutation (Table 1), we focused our efforts on studying the properties of the Glu352Gln^Sd^ protein.

As the presence of an acyl moiety at position-350 was only inferred from hydroxylamine treatments, we decided to experimentally confirm that Lys350^Sd^ is indeed acylated in Glu352Gln^Sd^ before studying the properties of this mutant protein. Peptide mapping experiments were carried out (Fig. S2). The results show that the mass of the peptide corresponding to the region 346–357, Sd^346–357^, is increased by the size of an adduct corresponding to a palmitate/myristate, while the mass of the peptide corresponding to the region 370–382, Sd^370–382^, matches that of the unmodified peptide. In contrast, Sd^346–357^ is unmodified and Sd^370–382^ is acylated in Lys350Ala^Sd^. In line with the hydroxylamine treatment experiments, these results confirm that Lys350^Sd^ is acylated in Glu352Gln^Sd^.

### Effect of the acylation on the thermal stability of Sd

A Fluorescence Thermal Shift Assay (FTSA) was used to measure the thermal stability of the wild type and mutant proteins (Fig. S3). The thermograms obtained with wt^Sd^ and Lys350Ala^Sd^ show one peak (Fig. S3). The melting temperature (T_m_) of Lys350Ala^Sd^ (52 °C) is significantly lower than that of wt^Sd^ (58 °C; ΔT_m_ = T_m_^wt^ − T_m_^mutant^ = 6 °C, Table 1) although both proteins are fully acylated (Sup. Figure 1, but see below). The thermograms obtained with Glu352Gln^Sd^ show two peaks (Fig. S3), one with the same T_m_ as Lys350Ala^Sd^ (52 °C) and another one with a much higher T_m_ (63 °C, Table 1). Combining this result with the data obtained from the hydroxylamine treatments (Table 1), we propose that the peak with the lower T_m_ measures the unfolding of the protein acylated on Cys373^Sd^ while the one with the higher T_m_ measures the unfolding of the protein acylated on Lys350^Sd^. Therefore, Lys350^Sd^ acylation has a stronger stabilizing effect on Sd than Cys373^Sd^ acylation (ΔT_m_ ~ 11 °C). The analysis of the thermograms obtained with Glu352Gln^Sd^, Glu352Leu^Sd^ and wt^Sd^ shows that when the proportion of acyl-Cys373^Sd^ increases in the protein preparations, the two well-separated peaks observed with Glu352Gln^Sd^ progressively merge into a single peak as seen with wt^Sd^ (Fig. S3). Therefore, the single peak observed for wt^Sd^ does not measure the T_m_ of one species but of a mixture containing similar amounts of both acyl forms. The T_m_ of wt^Sd^ (58 °C) is thus intermediate between those of acyl-Cys373^Sd^ (52 °C) and acyl-Lys350^Sd^ (63 °C), explaining why Lys350Ala^Sd^, which contains only acyl-Cys373^Sd^, has a lower T_m_ than wt^Sd^.

### Interaction between Yki, Vg, Tgi, and Glu352Gln^Sd^

The residues of TEAD corresponding to Lys350^Sd^ and Glu352^Sd^ are located in a β-strand that interacts with a β-strand present in the TEAD-binding domain of YAP, VGLL1, and VGLL4 upon binding^17–19^. By homology, Yki, Vg (vestigial, *Drosophila* homolog of human VGLL1-3), and Tgi (Tondu domain containing growth inhibitor, *Drosophila* homolog of human VGLL4) should contain a β-strand in their Sd-binding domain that interacts with the β-strand where are present Lys350^Sd^ and Glu352^Sd^. Therefore, the Glu352Gln^Sd^ mutation and/or the acylation of Lys350^Sd^ may affect the interaction between these proteins and Sd. To check this possibility, the interaction between Yki^30–146^/Vg^263–382^/Tgi^2–382^ (which contain the Sd-binding domains of these proteins) and Glu352Gln^Sd^ was studied by Surface Plasmon Resonance (Fig. S4). Our results show that the Glu352Gln^Sd^ mutation does not significantly affect the interaction between Yki/Vg/Tgi and Sd because these proteins bind with a similar affinity to both wt^Sd^ and Glu352Gln^Sd^ (Table 2).

**Table 2:** Affinity of Yki/Vg/Tgi for wt^Sd^, Glu352Gln^Sd^ and Lys350Ala^Sd^.

Proteins	Yki^30–146^ (K_d_ nM)	Vg^263–382^ (K_d_ nM)	Tgi^2–382^ (K_d_^app^ nM)
wt^Sd^	9.5 ± 0.5	0.38 ± 0.02	570 ± 50; 3.0 ± 0.4
Glu352Gln^Sd^	4.9 ± 0.3	0.37 ± 0.02	550 ± 30; 2.8 ± 0.4
Lys350Ala^Sd^	14 ± 1	2.3 ± 0.3	910 ± 60; 6 ± 1

The affinity (K_d_) was measured by Surface Plasmon Resonance in experiments where the biotinylated-Avitagged-Sd proteins were immobilized on sensor chips. Two apparent K_d_ values, K_d_^app^, were obtained for Tgi because this protein contains two binding sites for Sd (for details see^28^). Averages and standard errors are given (n ≥ 2).

We also included the Lys350Ala^Sd^ mutant in these experiments. This protein has a similar affinity for Yki^30–146^ and Tgi^2–382^ as wt^Sd^ does (Table 2). However, we measured a sixfold change in binding affinity for Vg^263–382^ (Table 2). It is difficult to conclude whether this significant difference comes from the Lys350Ala^Sd^ mutation per se, the acylation of Cys373^Sd^, or a combination of both. Nevertheless, it is interesting to note that the β-strand region present in the TEAD-binding domain of VGLL1 (Vg)^20^, that binds near the acylation site, contributes more to the interaction with TEAD than the corresponding region in YAP (Yki) or VGLL4 (Tgi)^17,21^.

In summary, our biochemical data reveal that Sd can be acylated either on Cys373^Sd^ or on Lys350^Sd^. The Glu352Gln^Sd^ mutation enhances the level of acylation on Lys350^Sd^. Acylated Lys350^Sd^ has a higher thermal stability than acylated Cys373^Sd^. The Glu352Gln^Sd^ and wt^Sd^ proteins bind to Yki, Vg, or Tgi with a similar affinity.

### Acylation of Lys350^Sd^ in insect cells

Since Lys350^Sd^ acylation described above was observed for truncated forms of Sd (residues 223–440) expressed in *E. coli*, we checked whether it also occurs in the full-length Sd protein (Sd^fl^) expressed in insect cells. Despite multiple attempts, we were unable to obtain sufficient amounts of Sd^fl^ from transient transfections in *Drosophila* S2 cells. Therefore, we used cells derived from another insect species, *Spodoptera frugiperda* (Sf21 cells). The Sd^fl^ and Glu352Gln^fl^ proteins, which were successfully expressed and purified from *S. frugiperda*, were obtained as a mixture of non-covalently modified and covalently attached to myristic, palmitic (or stearic acid) forms (Fig. S1). The incubation of these mixtures with Myristoyl-Coenzyme A (Myr-CoA) leads to a complete acylation of these proteins, revealing that both proteins are able to undergo an auto-acylation reaction (Fig. S1). The analysis of the reaction between the non-Myr-CoA treated proteins and hydroxylamine shows that the fraction of protein which is resistant to hydroxylamine is significantly higher with Glu352Gln^fl^ than with Sd^fl^. This is also true for the proteins that have been pre-treated with Myr-CoA (Fig. S1). Altogether, these results suggest that Sd^fl^ is acylated on Lys350^Sd^ when expressed in insect cells, and that this acylation is enhanced by the Glu352Gln^Sd^ mutation.

To confirm that Glu352Gln^fl^ expressed in *S. frugiperda* is indeed acylated on Lys350^Sd^, we looked for the presence of a covalent modification of this residue by mass spectrometric analysis of tryptic digests of the proteins. This analysis confirms the presence of a myristate/palmitate modification on Sd^346–357^ while Sd^370–382^ is unmodified (Fig. S2). Sd^fl^ was also included in these experiments and both peptides are acylated in this protein. Altogether, our findings show that Glu352Gln^fl^ purified from insect cells is largely acylated on Lys350^Sd^, while Sd^fl^ is acylated on both Lys350^Sd^ and Cys373^Sd^.

### In vivo study of the Glu352Gln^Sd^ mutant

To gain more insight into a potential role of Lys350^Sd^ acylation on Sd function in vivo, we decided to study the biological consequences of the expression at physiological levels of Glu352Gln^Sd^ in *Drosophila*. In contrast to mammals, in which four TEAD genes are present, *Drosophila* only contains one copy, *Sd*, making it an ideal model organism to study the regulation of these transcription factors. Therefore, we decided to introduce the Glu352Gln^Sd^ mutation into the endogenous *sd* locus, shifting the acylation state of Sd towards Lys350^Sd^.

**Figure 2:**
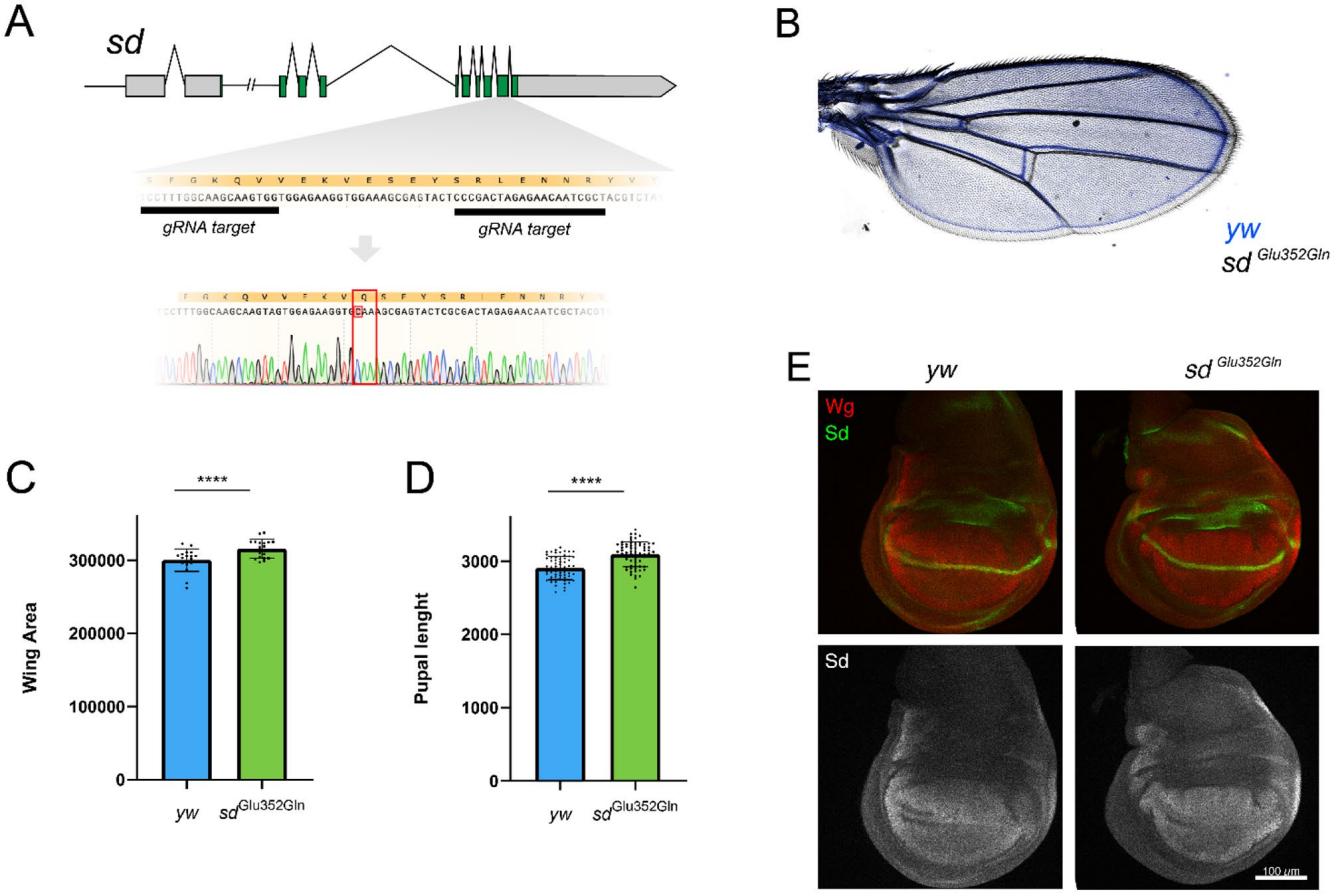
Generation of Glu352Gln^Sd^ flies and in vivo analysis. (**A**) Structure of *sd* gene in *Drosophila*. Spacer sequence of gRNAs in highlighted, along with the nucleotide and putative amino acid sequence. Below, Sanger sequencing results of the Glu352Gln^Sd^ mutant. Notice that some silent mutations are introduced in order to avoid donor plasmid cutting by the gRNAs (grey points). (**B**) Superimposed wt^Sd^ (blue) and Glu352Gln^Sd^ mutant (grey) wings. (**C**) Quantification of wing area in arbitrary units, showing a small (5.2%) but significant increase in wing area in Glu352Gln^Sd^ mutants. P value < 0.0001 (wt^Sd^ n = 18, Glu352Gln^Sd^ n = 18). (**D**) Quantification of pupal size (6.8% difference). P value < 0.0001 (wt^Sd^ n = 60, Glu352Gln^Sd^ n = 64). (**E**) Immunostaining of Sd and Wg proteins in third instar imaginal wing discs. The Glu352Gln^Sd^ mutation does not result in a noticeable change of Sd levels.

In order to generate the Sd mutant, we used ad hoc CRISPR techniques, involving the introduction of a marker and its subsequent seamless removal (a manuscript describing the method in detail is in preparation). After removing the marker, the locus was sequenced to ensure seamless introduction of the correct mutation (Fig. 2A). Homozygous mutants reach adulthood, are fertile and do not present obvious morphological defects. In order to enhance putative phenotypes, and bearing in mind the higher thermal stability of Lys350^Sd^ compared to wild type, we performed all subsequent analyses at 29 °C. We noticed a slight increase in adult size (exemplified by wing area) (Fig. 2B,C) as well as in pupal size (Fig. 2D). In order to correct for possible crowdedness or food accessibility differences, we incubated both genotypes (wild type and mutant) in the same glass tube, resulting in the same differences (Fig. S5). We evaluated whether Sd protein levels were different in the Glu352Gln^Sd^ mutant and found that, at least in imaginal wing discs, wild type Sd levels are comparable to those of the mutant protein (Fig. 2E). We were also unable to detect obvious changes in the transcriptional output of Sd in late-third instar wing disc using a LacZ reporter of the Sd target *expanded* (*ex*) (Fig. S6). Together, our results suggest that increasing Lys350^Sd^ acylation modestly affects the growth control mediated by Sd, but it was not possible to determine whether this was due to enhanced target activity or an indirect mechanism.

## Discussion

The acylation of the TEAD/Sd transcription factors is important for the function of these proteins^10,11,13^. It has already been established that this post-translational modification takes place on a conserved cysteine, but we show here that Sd can be acylated either on Cys373^Sd^ or on Lys350^Sd^. The proximity of these two residues in the structure of Sd, and the absence of acylation of the TEAD/Sd proteins when the cysteine is mutated^10–12^, suggest that Lys350^Sd^ becomes acylated following the transfer of the acyl moiety from the cysteine residue. Since recombinant Sd purified from *E. coli* is a mixture of proteins acylated on Cys373^Sd^ or on Lys350^Sd^, we designed the Glu352Gln^Sd^ mutation to enhance Lys350^Sd^ acylation. The Glu352Gln^Sd^ protein has a higher thermal stability than the Lys350Ala^Sd^ protein (acylated only on Cys373^Sd^) and it binds to Yki, Tgi, and Vg with the same affinity as wt^Sd^. As the Glu352Gln^Sd^ mutation also enhances Lys350^Sd^ acylation in full-length Sd expressed in insect cells, we introduced this mutant into *Drosophila* to study the effect of Lys350^Sd^ acylation on the function of Sd in an in vivo context.

Our results show that the mutant flies are viable and do not present morphological defects. Furthermore, the Glu352Gln^Sd^ and the wild type proteins have a similar level and pattern of expression in flies. In line with the biochemical data, these in vivo findings indicate that the Glu352Gln^Sd^ mutation does not alter significantly the functionality of Sd. Therefore, the enhanced acylation of Lys350^Sd^ triggered by this mutation is well tolerated during *Drosophila* development, during which the Hippo/Salvador pathway plays a critical role^22,23^. We also noticed that the pupal and adult sizes of the mutant flies are statistically larger than the wild type ones. The magnitude of this difference is rather small and it cannot be totally excluded that it results from other mutation(s) in the chromosome that escaped our investigations. Nevertheless, we know from our biochemical data that wt^Sd^ is also acylated on Lys350^Sd^. Therefore, a larger difference in size might have been measured if the Glu352Gln^Sd^ flies were not compared to wild type flies, but to flies where Sd is only acylated on Cys373^Sd^, such as flies expressing the Lys350Ala^Sd^ mutant. This is exemplified by the results from the fluorescence thermal shift assay where we measured a larger difference in melting temperature between Glu352Gln^Sd^ and Lys350Ala^Sd^ (ΔT_m_ = 11 °C) than between Glu352Gln^Sd^ and wt^Sd^ (ΔT_m_ = 5 °C). Unfortunately, since the Lys350Ala^Sd^ mutation reduces the affinity of Sd for Vg, differences between Glu352Gln^Sd^ and Lys350Ala^Sd^ flies may come from the acylation of Sd on two different residues, the different affinities of the two mutant proteins for Vg, or from a combination of both effects making any interpretation very difficult. In the absence of experimental data, we just can hypothesize that acyl-Lys350^Sd^, which has a higher thermal stability and is insensitive to thioesterases, forms a more stable Sd pool in cells than acyl-Cys373^Sd^ that has a lower thermal stability and is sensitive to thioesterases^13^. This may explain the decreased stability of TEAD observed in cells when the lysine corresponding to Lys350^Sd^ is mutated to alanine^11^. If our hypothesis is correct, these two pools of Sd/TEAD should have overlapping but also distinct behaviors in cells and the small increase in size of the Glu352Gln^Sd^ flies when compared to the wild type flies might be triggered by the expression of the more stable acyl-Lys350^Sd^ pool in the mutant flies.

The finding that TEAD/Sd can be acylated on a lysine may also have implications beyond the study of the role of this post-translational modification on the function of these proteins. The TEAD transcription factors are currently being investigated extensively in oncology and compounds which bind to these proteins are being tested as novel anticancer agents^24^. Several of these molecules target the myristate/palmitate-binding pocket of TEAD, either making a covalent adduct with the cysteine or binding to this site in a non-covalent manner^25–27^. Since the stability of TEAD is enhanced by the acylation of the lysine, the fraction of acyl-Lys TEAD present in tumor cells could be more resilient to treatments by such compounds.

## Material and methods

### Expression in *Escherichia coli*

Sd^223–440^ (wt^Sd^), Yki^30–146^, Vg^263–382^ and Tgi^2–382^ were obtained as previously reported^21,28^. The Lys350Ala^Sd^, Glu352Gln^Sd^ and Glu352Leu^Sd^ mutations were introduced into wt^Sd^ using a previously-described methodology^29^ and the corresponding proteins were expressed/purified in a similar manner to wt^Sd^. As a significant fraction of the purified Sd proteins is not acylated, the proteins were treated with Myristoyl-Coenzyme A to reach complete acylation^12^. This acylation step enables the use of more homogeneous material in the biochemical/biophysical assays. The purity and the molecular weight of the proteins were assessed by LC–MS (Fig. S1).

### Expression in *Spodoptera frugiperda*

Cloning and expression. DNA encoding full-length Sd was obtained from GeneArt (Thermo Fisher, Switzerland). The DNA was extended by LguI recognition site adaptors and subcloned by T2S restriction enzyme cloning^30^ into a modified pFastBac1 vector providing an N-terminal His_10_-tag, a ZZ-solubilizing-tag and a glycine spacer, followed by an HRV3C protease cleavage site. In brief, the vector and the synthetic DNA insert were incubated in buffer B (Fermentas, Waltham, MA) supplemented with 1 mM ATP, 1 mM DTT, 5 U LguI, and 5 U T4 DNA ligase for 30 cycles (30 min at 37 °C, 1 min at 10 °C, 1 min at 30 °C). The mutation Glu352Gln^Sd^ was introduced as previously described^29^. The final expression constructs were confirmed by Sanger sequencing. Recombinant baculoviruses were generated using the Bac-to-Bac protocol (Invitrogen, Carlsbad, CA). The baculovirus stocks were amplified once and frozen following the Titerless Infected-cells Preservation and Scale-up protocol (TIPS^31^). Large-scale expressions were conducted in Sf21 cells (1 × 10^6^ cells/mL) for 96 h. Protein purification. Insect cell pellets were resuspended in buffer A (50 mM Tris, 300 mM NaCl, 20 mM imidazole, 1 mM TCEP, 10% glycerol, pH 8.0) supplemented with 0.2% v/v Triton X-100, Turbonuclease (final concentration 40 units/mL) and cOmplete protease inhibitor tablets (1 tablet/ 50 mL). The cells were lysed by two rounds of sonication for 40 s each with a Vibra Cell equipped with tapered probe (Sonics, Newtown, CT). The lysates were centrifuged at 40000xg for 40 min (Sorvall Lynx F20-12 × 50 rotor, ThermoFisher Scientific, Waltham, MA) and the supernatant was passed through a 0.45 μm filter. The cleared lysates were then loaded onto a 5 mL HisTrap FF Crude column (Cytiva, Marlborough, MA) mounted on an ÄKTA Pure 25 chromatography system (Cytiva, Marlborough, MA). Unbound material was washed away with buffer A. Bound protein was eluted with a 0–100% linear gradient of buffer B (buffer A containing 300 mM imidazole). HRV 3C protease (His-MBP-tagged, produced in-house) was added to the eluted protein (~ 1:100 w/w ratio). The N-terminal purification tag was cleaved off by the protease during dialysis overnight at 5 °C against 1 L buffer A. The protein solution was loaded again onto the HisTrap FF column and the flow-through containing the target protein was collected. The protein was concentrated with an Amicon Ultra-15 concentrator (MWCO 10,000, Merck, Germany) and injected onto a ProteoSEC 16/60 6–600 HR size exclusion column (ProteinArk, UK) pre-equilibrated with 50 mM Tris pH 8.0, 100 mM NaCl, 2 mM MgCl_2_, 1 mM TCEP, 5% glycerol. Fractions containing pure protein were pooled and frozen on dry ice. The purity and concentration of the protein was determined by RP-HPLC; its identity was confirmed by LC–MS.

### Hydroxylamine treatments

The proteins (0.2–0.50 mg/mL) were incubated for 60–90 min at room temperature in the presence of 50 mM hydroxylamine (Sigma-Aldrich, St Louis, MI) in 50 mM HEPES pH 7.4, 100 mM KCl, 0.25 mM TCEP, and 1 mM EDTA. At the end of the reaction, the de-acylation of the protein was assessed by analytical RP‐UHPLC and LC–MS. Samples were analyzed using an ACQUITY BEH300 C4 2.1 × 100 mm 1.7 μm column (Waters, Milford, MA) in a Nexera-I system (Shimadzu, Japan) using a 20–70% gradient of water/acetonitrile (+ 0.1% trifluoroacetic acid) at 80 °C. Elution of the protein was monitored by its absorbance at 210 nm. Intact protein mass was measured by a Xevo‐G2‐XS QTof mass spectrometer coupled to an ACQUITY UPLC system (Waters, Milford, MA). Representative chromatograms are represented on Fig. S1.

### Fluorescence thermal shift assay and surface plasmon resonance

The Fluorescence Thermal Shift Assay and Surface Plasmon Resonance experiments were carried out as previously described^28^. Representative thermograms and sensorgrams are represented on Fig. S3 and S4, respectively.

### Peptide mapping

For in-solution digestion without reduction and alkylation (to avoid loss of modification) the diluted proteins (3–9 μg, 3–10 µL) were incubated for 5 min at room temperature with an equivalent volume of 8 M urea, 0.5 M Tris pH 8.4. A solution (1 µL) containing 1 M Tris pH 10.0 and 1 µg of Trypsin/LysC (Promega, Madison, WI) was added to the protein solutions and the mixtures were diluted in water (final volume 100 µL) prior an overnight incubation at 37 °C, under constant shaking (500 rpm). The samples were next acidified with 1% trifluoro acetic acid to stop the digestion. The resulting peptide mixtures were desalted using a ZipTip C18 column (Millipore, Burlington, MA) and dried before reconstitution in 0.1% formic acid in water. The digested samples (100 ng) were analyzed by LC-MSMS on a Q-Exactive HF-X mass spectrometer equipped with an Easy-nanoLC system (Thermo Fisher Scientific, Waltham, MA). Samples were injected onto a ReproSil-Pur C18 column (5 cm × 100 µm ID; 3 µm beads) (Evosep Biosystems, Denmark) and eluted with a 40 min gradient from 0 to 50% acetonitrile in 0.1% formic acid. Mass spectrometric analysis was performed using a top 10 data-dependent acquisition method using the following settings. MS1 resolution 120,000 with a maximum injection time of 50 ms and an AGC target of 3E6 ions. MSMS resolution 15,000 with a maximum injection time of 50 ms, an AGC target of 1E5 ions, a stepped normalized collision energy setting of 27, 29, 31. We found that the peptide match setting had to be set ‘off’ for reliable triggering of MS2 scans on the modified peptides, probably because their isotope pattern deviates from the expected pattern for peptides. Raw files were processed using the Mascot search engine (v. 2.6.0; Matrix Science, UK) using myristoylation (+ 210.198 Da) and palmitoylation (+ 238.230 Da) as variable modifications on cysteine and lysine residues.

### Generation and analysis of the Glu352Gln^Sd^ mutant in *Drosophila*

Generation of Glu352Gln^Sd^. To generate Glu352Gln^Sd^ mutants, a template donor bearing the desired mutation was co-injected with gRNA-expressing plasmids into flies expressing Cas9 under *nos* promoter (Bloomington stock number 78782). Flies were screened for the presence of a marker that was subsequently removed. Mutants were confirmed by Sanger sequencing. Flies were outcrossed with *y,w* to remove *nos-Cas9* and possible off-target mutations. gRNAs were cloned in the pCFD5 plasmid following published methods^32^. The gRNA spacer sequences used were: 5′-TTGCTCCTTTGGCAAGCAAG-3′ and 5′-CGTAGCGATTGTTCTCTAGT-3′. The outer most primers of the homology arms were: 5′-GGCAAATATTCGACAAGTTTCCGGAG-3′ and 5′-TGTGAACTTGTGTGTTGATGATGGG-3′, resulting in homology arms of 400 base pairs each. Analysis of the flies. Flies were kept in regular corn-based food with a drop of yeast. Only tubes with similar numbers of flies were compared. To compare different genotypes in the same tube, inseminated female flies of similar ages were mixed and left to lay eggs for 1 day. After blind quantification, flies were sequenced and their genotype assigned. The immunostaining was performed as follows: third instar larvae were dissected to expose the wing imaginal discs in cold PBS. Immediately, samples were transferred to fixing solution (4% PFA (C993M31, Electron Microscopy Sciences, Hatfield, PA) in PBS) and incubated for 30 min at room temperature. After thoroughly washing with PBS, samples were permeabilized with PBST (0.3% Triton X-100 in PBS) and then blocked in 5% NGS (ab7481, Abcam, Switzerland) in PBT for 1 h. Incubation with primary antibody diluted in blocking solution was subsequently performed over-night at 4 °C. Samples were then washed with PBST and incubated in secondary antibody diluted in blocking solution. After washing with PBST and PBS, samples were mounted for confocal imaging in Vectashield (H-1200, Vector Laboratories, Burlingame, CA). The primary antibodies used in this study were: guinea pig anti-Sd (gift from Prof. Guss, Dickinson College, PA), mouse anti-Wg (clone 4D4 DSHB, University of Iowa, Iowa City, IA) and anti-βGal (Abcam, ab9361). The secondary antibodies used were: goat anti-mouse Alexa568, goat anti-guinea pig Alexa488 and anti-chicken Alexa 488 (Thermo Fisher Scientific, Waltham, MA). Samples were imaged using a Zeiss LSM880 point confocal microscope. *ex-LacZ*^*697*^ strain was obtained from Bloomington stock center. The changes in the different wing area and pupal length were analyzed in PRISM by unpaired t-tests.

## Supplementary Information

Supplementary Information.
