# Supplementary material for: The role of lysine palmitoylation/myristoylation in the function of the TEAD transcription factors
Source: Sci Rep. 2022 Mar 23;12:4984. doi: 10.1038/s41598-022-09127-7 (PMC8942982; doi:10.1038/s41598-022-09127-7)

Supplementary Information

The role of lysine palmitoylation/myristoylation in the function of the TEAD transcription factors

Yannick Mesrouze[1#], Gustavo Aguilar[2#], Marco Meyerhofer[1], Fedir Bokhovchuk[1], Catherine Zimmermann[1], Patrizia Fontana[1], Alexandra Vissières[3], Hans Voshol[3], Dirk Erdmann[1], Markus Affolter[2] & Patrick Chène[1*].

[1] Disease Area Oncology, Novartis Institutes for Biomedical Research, Novartis, 4002 Basel, Switzerland.

[2] Biozentrum, University of Basel, Spitalstrasse 41, 4056 Basel, Switzerland.

[3] Analytical Sciences and Imaging, Novartis Institutes for Biomedical Research, Novartis, 4002 Basel, Switzerland.

[#] These authors contributed equally: Yannick Mesrouze and Gustavo Aguilar.

Figure S1. LC-MS analysis of the recombinant proteins.

Figure S2. Peptide mapping.

Figure S3. Fluorescence Thermal Shift Assay.

Figure S4. Surface Plasmon Resonance

Figure S5. Wing size comparison of flies raised in the same tube.

Figure S6. *Ex*-LacZ reporter expression in wt^{Sd} and $Glu352Gln^{Sd}$ backgrounds.

Figure S1. LC-MS analysis of the recombinant proteins. The figure shows HPLC profiles of representative protein preparations. For wt^{Sd}, $Lys350Ala^{Sd}$, $Glu352Gln^{Sd}$, $Glu352Leu^{Sd}$, Sd^{fl}, and $Glu352Gln^{fl}$ the left and right panels show chromatograms of the purified proteins before and after hydroxylamine treatment, respectively. Ac: acylated protein, n-Ac: non-acylated protein. The double peaks observed for the acylated proteins correspond to the myristoylated and palmitoylated variants of these proteins. The double peaks observed for the non-acylated proteins after hydroxylamine treatment correspond to the non-acylated protein and to the non-acylated protein with a hydroxylamine adduct (molecular weight Sd + 31).

Figure S2. Peptide mapping. **A**. $Glu352Gln^{Sd}$. MS/MS spectrum of parent ion mass 831.48 (2+) corresponding to the peptide QVVEKVQSEYSR myristoylated on $Lys350^{Sd}$. **B**. $Lys350Ala^{Sd}$. MS/MS spectrum of parent ion mass 907.97 (2+) corresponding to myristoylated peptide SPMCEYMINFIQK on $Cys373^{Sd}$. **C**. wt^{fl}. MS/MS spectrum of parent ion mass 831.97 (2+) corresponding to myristoylated peptide QVVEKVESEYSR on $Lys350^{Sd}$. **D**. wt^{fl}. MS/MS spectrum of parent ion mass 907.97 (2+) corresponding to myristoylated peptide SPMCEYMINFIQK on $Cys373^{Sd}$. **E**. $Glu352Gln^{fl}$. MS/MS spectrum of parent ion mass 831.48 (2+) corresponding to myristoylated peptide QVVEKVQSEYSR on $Lys350^{Sd}$. *: Further evidence for myristoylation on $Cys373^{Sd}$ is provided by neutral loss of the myristoyl residue resulting in the marked fragment masses in Fig. S2B and S2D. Labeling of the peptide fragment ions follows the standard nomenclature [1]. As expected with HCD fragmentation, y- and b-series ions are the dominating species. For each peptide, the sequence is confirmed by matching precursor and fragment masses against a custom sequence database, containing the expected Sd sequences. The myristoylated peptide SPMCEYMINFIQK was not detected in $Glu352Gln^{Sd}$ or $Glu352Gln^{fl}$, probably because of the low amount of acyl-$Cys373^{Sd}$ present in these samples (less than 10% of the total amount of the protein divided in two fractions Myr-$Cys373^{Sd}$ and Palm-$Cys373^{Sd}$).

Figure S3. Fluorescence Thermal Shift Assay. The figure shows representative thermograms obtained in the presence of 1 μM protein. The melting temperatures (T_m, curve minimum) were obtained by plotting the first derivative of the fluorescence emission (F) as a function of the temperature (–dF/dT).

Figur S4. Surface Plasmon Resonance. **A**, **D** and **G**. The figure represent sensorgrams obtained with Yki^{30-146} (left panel) and the corresponding binding isotherms (right panel) from which K_d values (at equilibrium) were derived. The sensorgrams were globally fitted with a 1:1 interaction model using the Biacore T200 evaluation software (Cytiva, Marlborough, MA). **B**, **E** and **H**. The affinity of $Vg^{263-382}$ was determined by Single Cycle Kinetic using the Biacore T200 evaluation software (Cytiva, Marlborough, MA). The maximum theoretical signal $R_{max}{}^{th}$ was calculated as follows: $R_{max}{}^{th} = (MW^{lig}/MW^{Sd}) \times R_{Sd} \times n$. MW^{lig} is the molecular weight of Yki^{30-146} or $Vg^{263-382}$. MW^{Sd} is the molecular weight of wt or mutant $Sd^{223-440}$. R_{Sd} is the level of immobilization of wt or mutant Sd in response unit (RU) and n = 1 is the stoichiometry of the interaction. **C**, **F** and **I**. The figures represent the sensorgrams obtained with Tgi^{2-382}. The affinity of the two binding sites present on Tgi, $K_d{}^{app}$, has been determined as already described [2].

Figure S5. Wing size comparison of flies raised in the same tube. Units are arbitrary. P-value <0.0001 (wt^{Sd} n=10, $Glu352Gln^{Sd}$ n=44).

Figure S6. *Ex*-LacZ reporter expression in wt^{Sd} and $Glu352Gln^{Sd}$ backgrounds. Immunostaining detection of β-Galactosidase (green) and Wg (red) in late-third instar wing discs. No noticeable change in the overall pattern or intensity is appreciated.

References

1 Biemann K. Contributions of mass spectrometry to peptide and protein structure. *Biomed Environ Mass Spectrom.* **16**, 99-111 (1988).

2. Mesrouze Y. et al. Biochemical properties of VGLL4 from *Homo sapiens* and Tgi from *Drosophila melanogaster* and possible biological implications. *Protein Sci.* **30**, 1871-81, (2021).

Figure S1

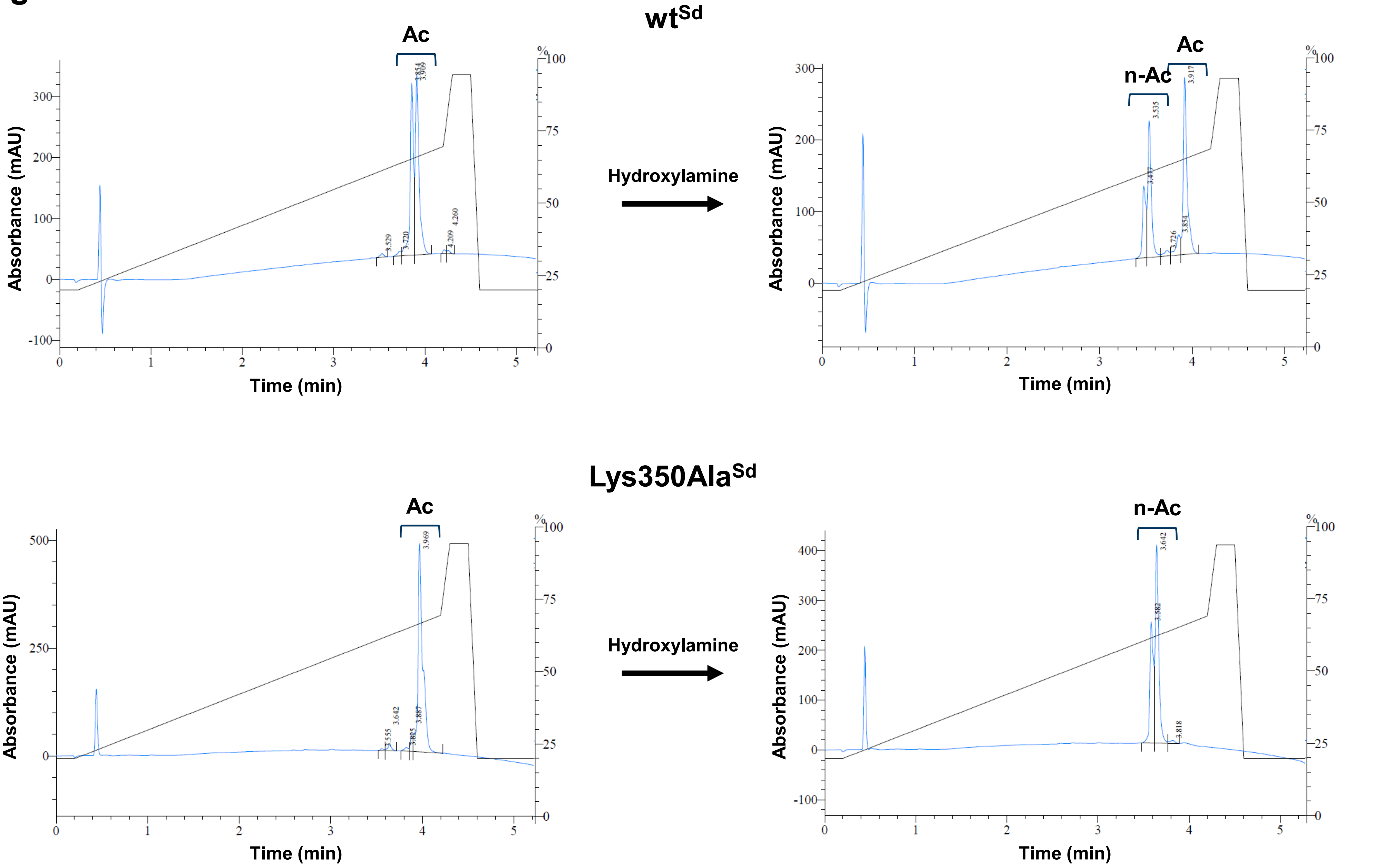

wt^Sd
Ac
n-Ac
Hydroxylamine
Lys350Ala^Sd
Absorbance (mAU)
Time (min)
%
3.854
3.909
3.529
3.720
4.209
4.260
3.535
3.917
3.472
3.726
3.969
3.555
3.642
3.825
3.887
3.582
3.818

Figure S1

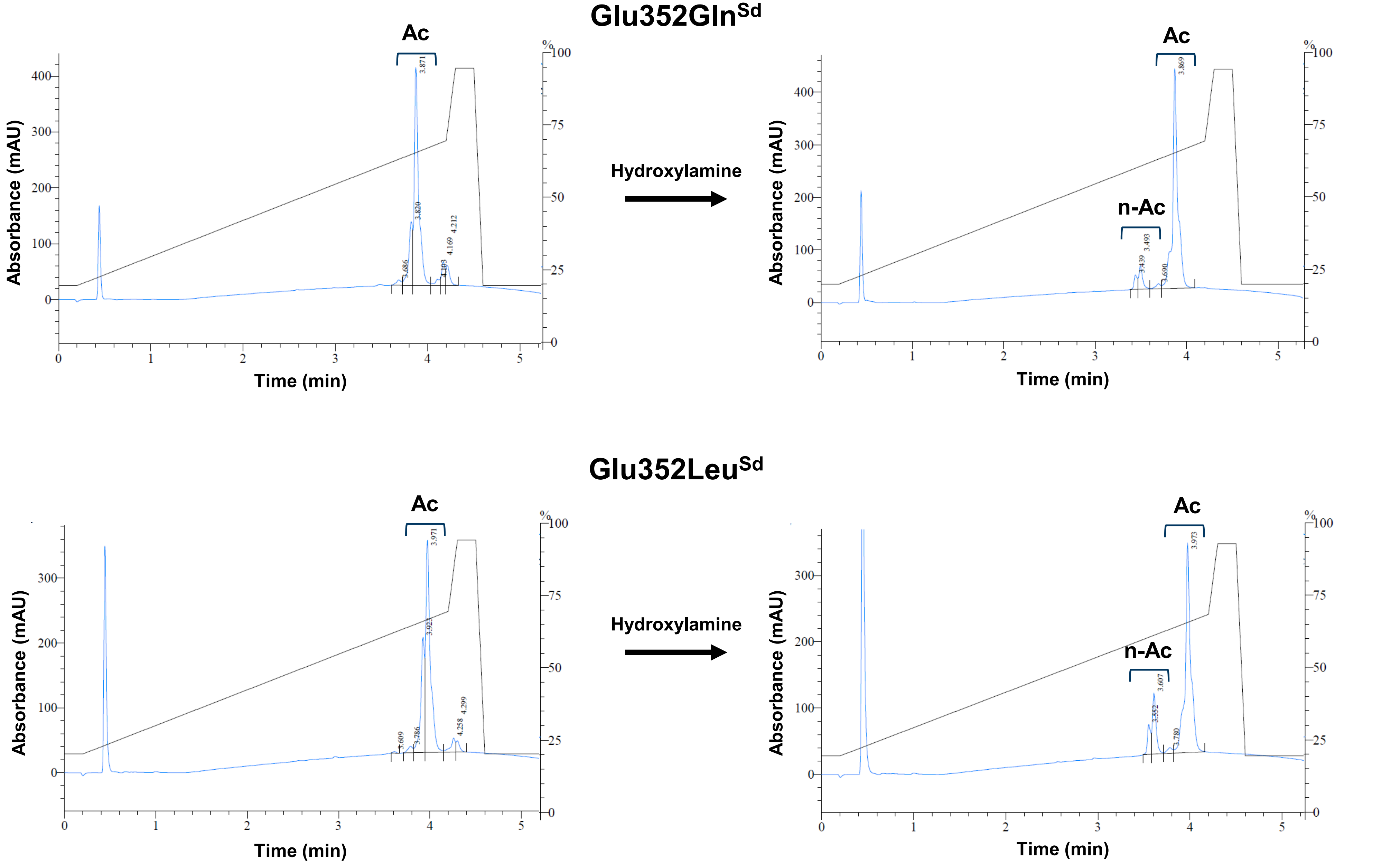

Glu352Gln^Sd
Ac
Hydroxylamine
n-Ac
Absorbance (mAU)
Time (min)
Glu352Leu^Sd

Figure S1

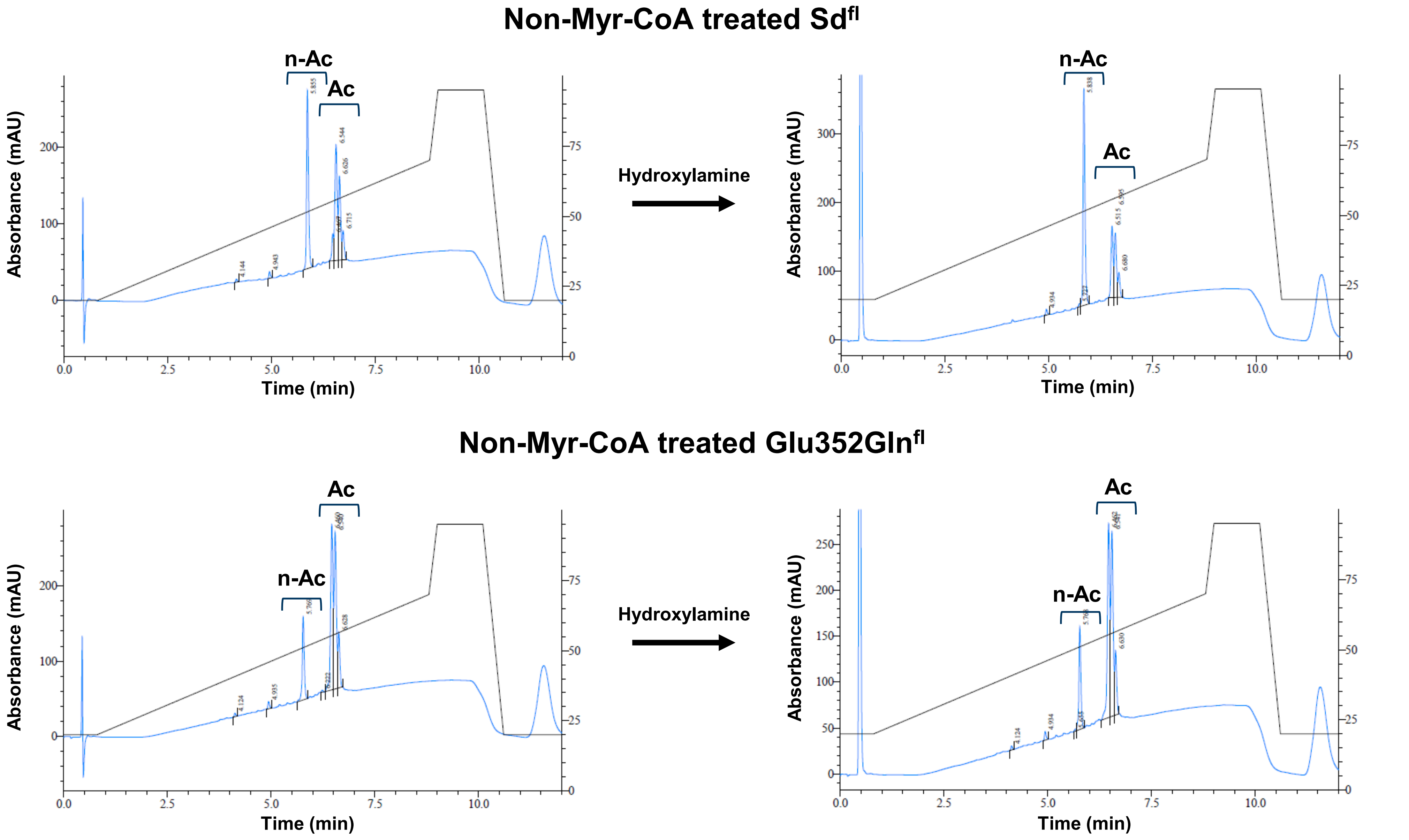

Non-Myr-CoA treated Sd^{fl}
n-Ac
Ac
Absorbance (mAU)
Time (min)
Hydroxylamine
Non-Myr-CoA treated Glu352Gln^{fl}
Ac
n-Ac
Absorbance (mAU)
Time (min)
Hydroxylamine

Figure S1

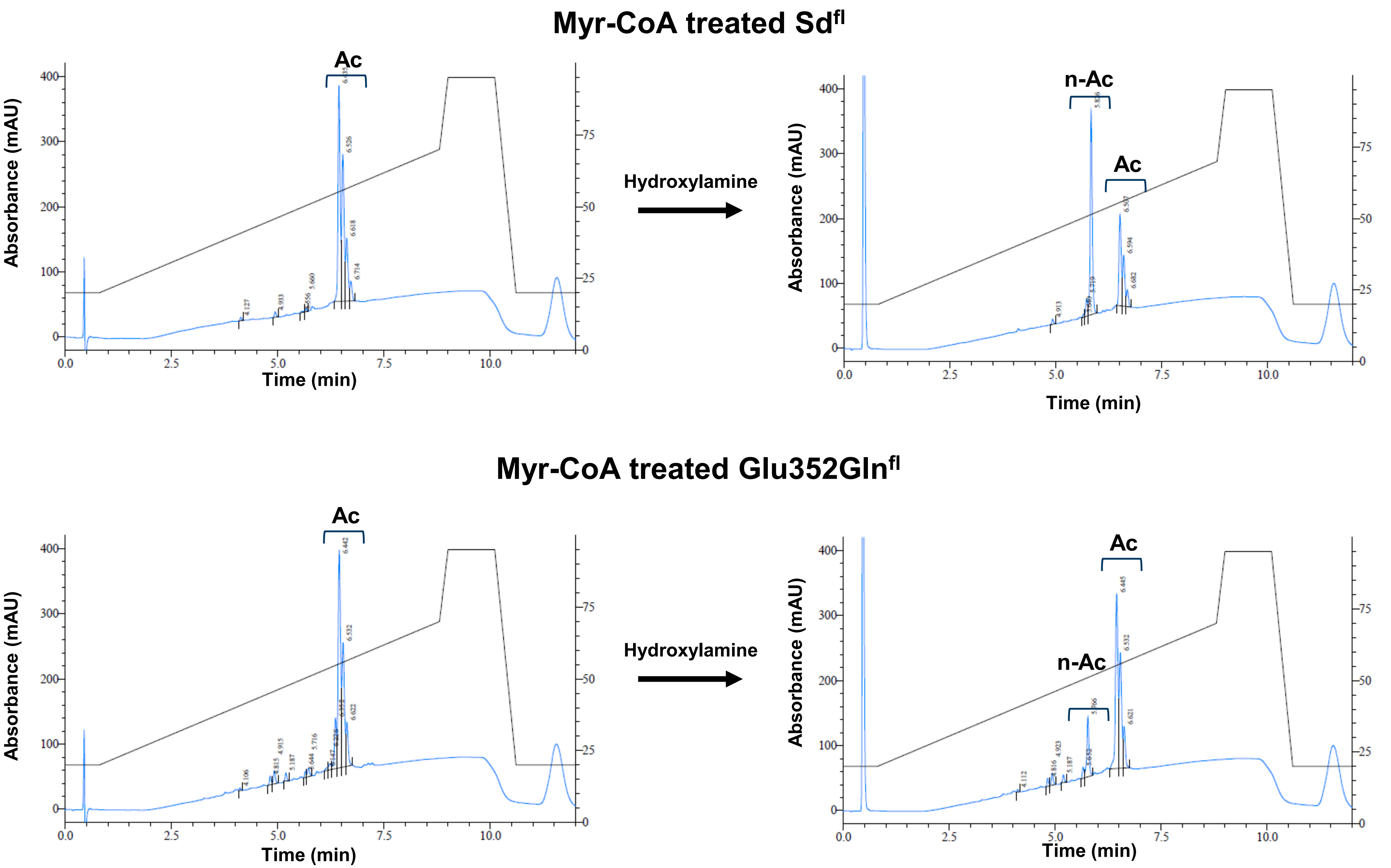

Myr-CoA treated Sd^{fl}
Ac
n-Ac
Ac
Hydroxylamine
Absorbance (mAU)
Time (min)
Myr-CoA treated Glu352Gln^{fl}
Ac
Ac
n-Ac
Hydroxylamine
Absorbance (mAU)
Time (min)

Figure S1

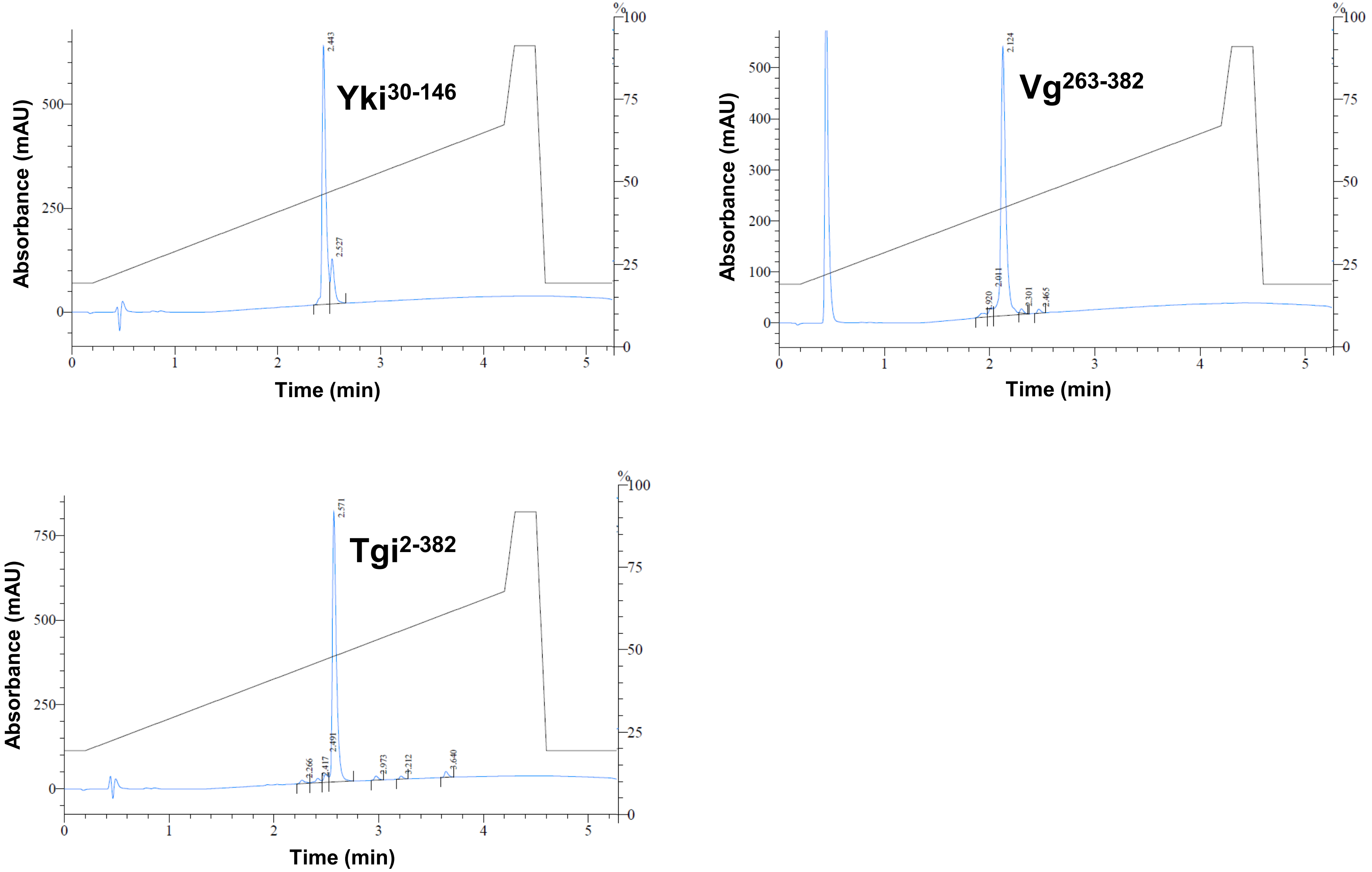

Yki$^{30-146}$
Vg$^{263-382}$
Tgi$^{2-382}$
Absorbance (mAU)
Time (min)
%
2.443
2.527
2.124
2.011
1.920
2.301
2.465
2.571
2.491
2.266
2.417
2.973
3.212
3.640

Figure S2

A

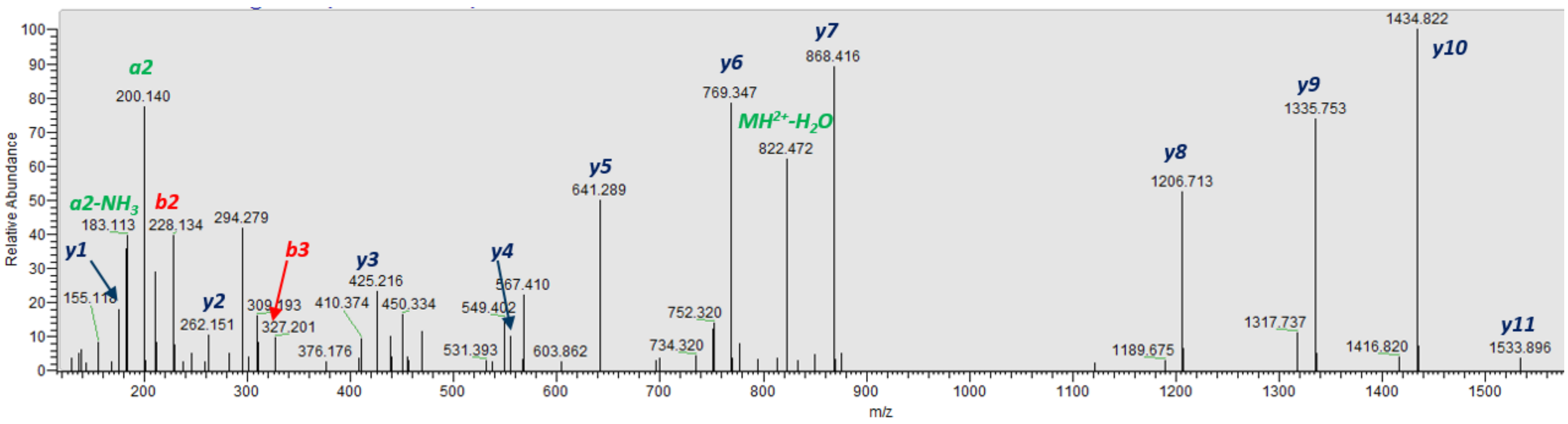

Relative Abundance
m/z
a2
200.140
a2-NH3
183.113
b2
228.134
294.279
y1
155.118
b3
309.193
327.201
y2
262.151
y3
425.216
410.374
450.334
376.176
y4
567.410
549.402
531.393
603.862
y5
641.289
752.320
734.320
y6
769.347
MH2+-H2O
822.472
y7
868.416
y8
1206.713
1189.675
y9
1335.753
1317.737
1434.822
y10
1416.820
y11
1533.896

B

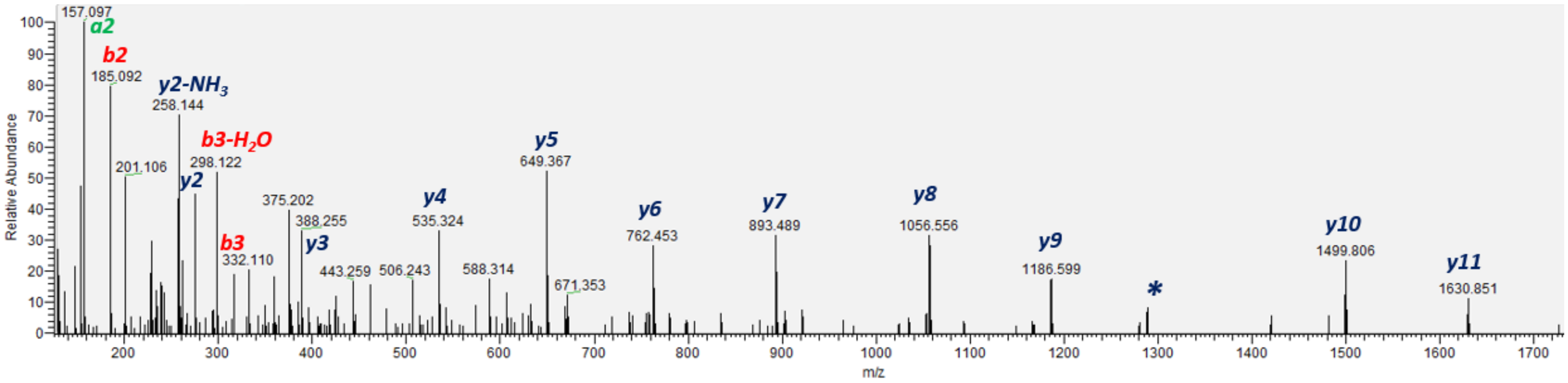

Relative Abundance
m/z
157.097
a2
b2
185.092
y2-NH3
258.144
201.106
y2
b3-H2O
298.122
b3
332.110
375.202
388.255
y3
443.259
506.243
y4
535.324
588.314
y5
649.367
671.353
y6
762.453
y7
893.489
y8
1056.556
y9
1186.599
*
y10
1499.806
y11
1630.851

Figure S2

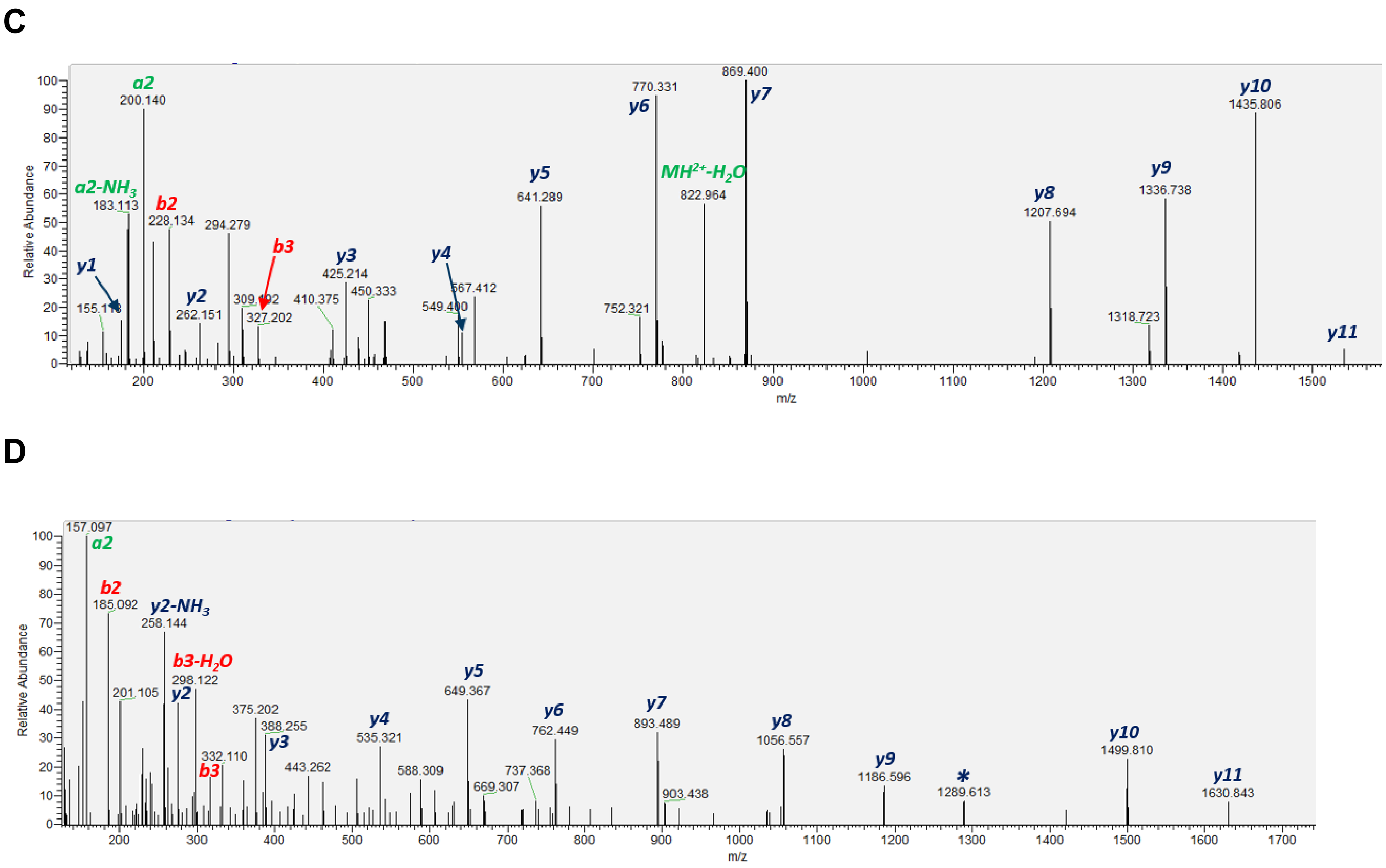
C
a2
200.140
a2-NH3
183.113
b2
228.134
294.279
y1
155.118
y2
262.151
309.192
b3
327.202
410.375
y3
425.214
450.333
y4
549.400
567.412
y5
641.289
752.321
770.331
y6
MH2+-H2O
822.964
869.400
y7
y8
1207.694
1318.723
y9
1336.738
y10
1435.806
y11
Relative Abundance
m/z
D
157.097
a2
b2
185.092
y2-NH3
258.144
201.105
b3-H2O
298.122
y2
375.202
388.255
y3
332.110
b3
443.262
y4
535.321
588.309
y5
649.367
669.307
737.368
y6
762.449
y7
893.489
903.438
y8
1056.557
y9
1186.596
*
1289.613
y10
1499.810
y11
1630.843
Relative Abundance
m/z

Figure S2

E

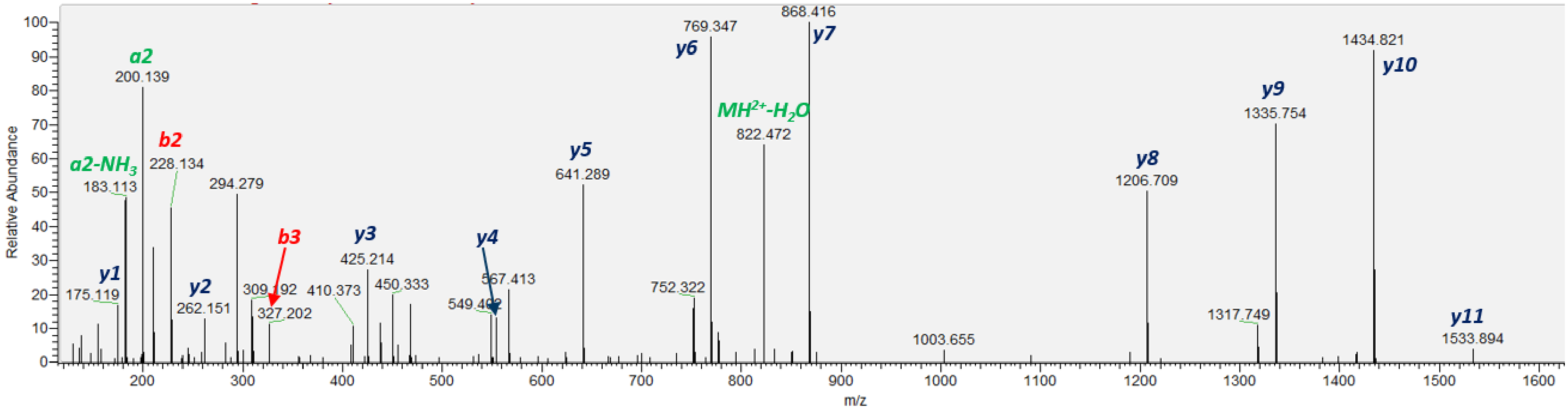

Relative Abundance
m/z
a2
200.139
b2
228.134
a2-NH3
183.113
294.279
y1
175.119
y2
262.151
309.192
b3
327.202
410.373
y3
425.214
450.333
y4
549.402
567.413
y5
641.289
752.322
y6
769.347
868.416
y7
MH2+-H2O
822.472
1003.655
y8
1206.709
y9
1335.754
1317.749
1434.821
y10
y11
1533.894

Figure S3

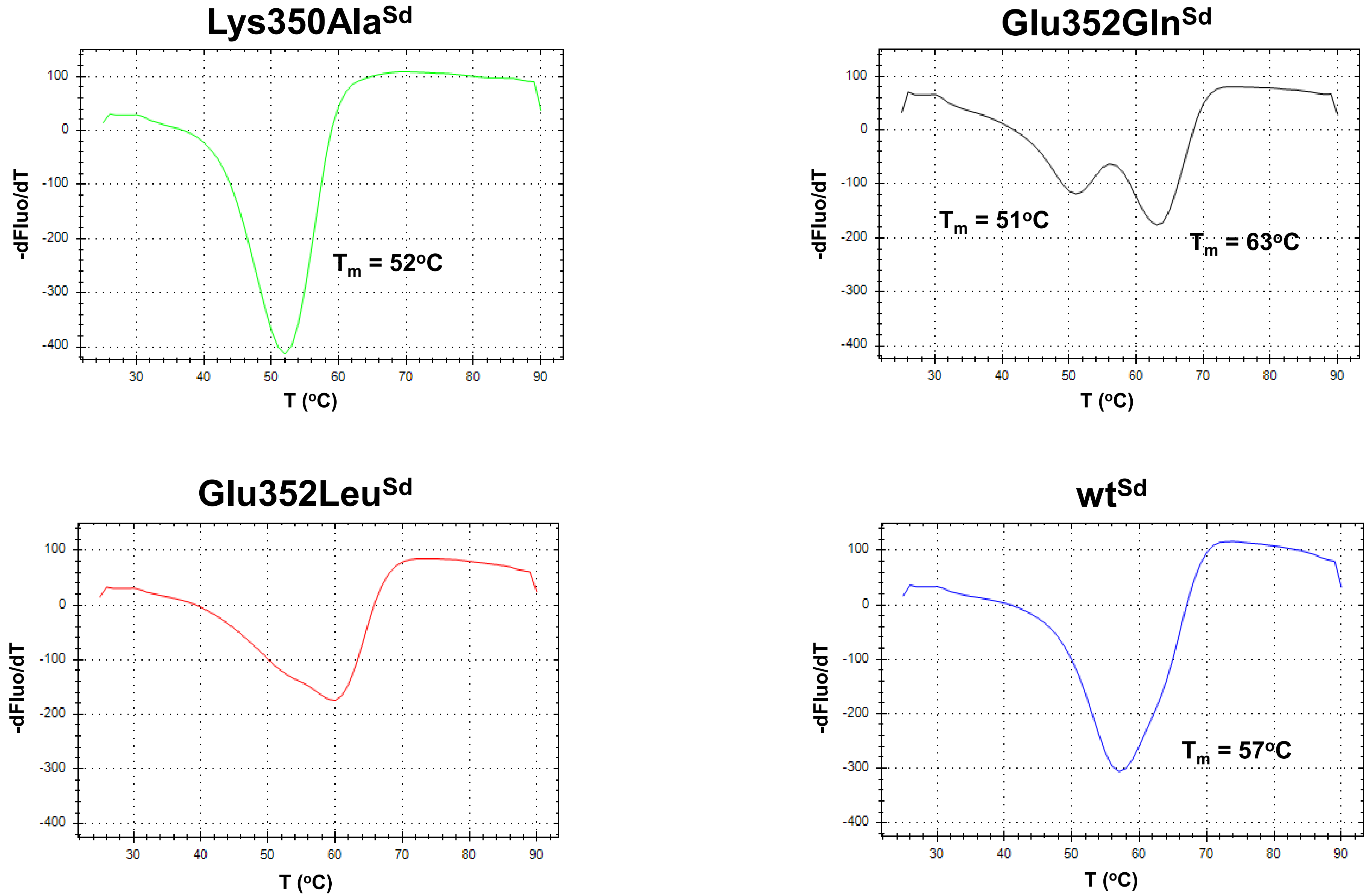

Lys350Ala^{Sd}
-dFluo/dT
T (°C)
T_m = 52°C
Glu352Gln^{Sd}
-dFluo/dT
T (°C)
T_m = 51°C
T_m = 63°C
Glu352Leu^{Sd}
-dFluo/dT
T (°C)
wt^{Sd}
-dFluo/dT
T (°C)
T_m = 57°C

Figure S4

A

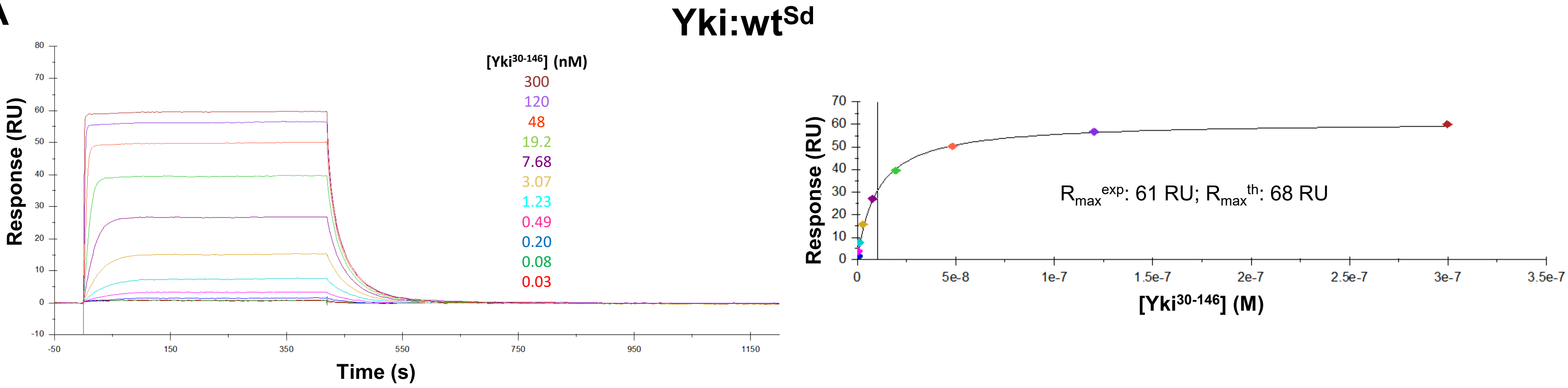

Yki:wtSd
[Yki30-146] (nM)
300
120
48
19.2
7.68
3.07
1.23
0.49
0.20
0.08
0.03
Response (RU)
Time (s)
Rmax exp: 61 RU; Rmax th: 68 RU
[Yki30-146] (M)

B

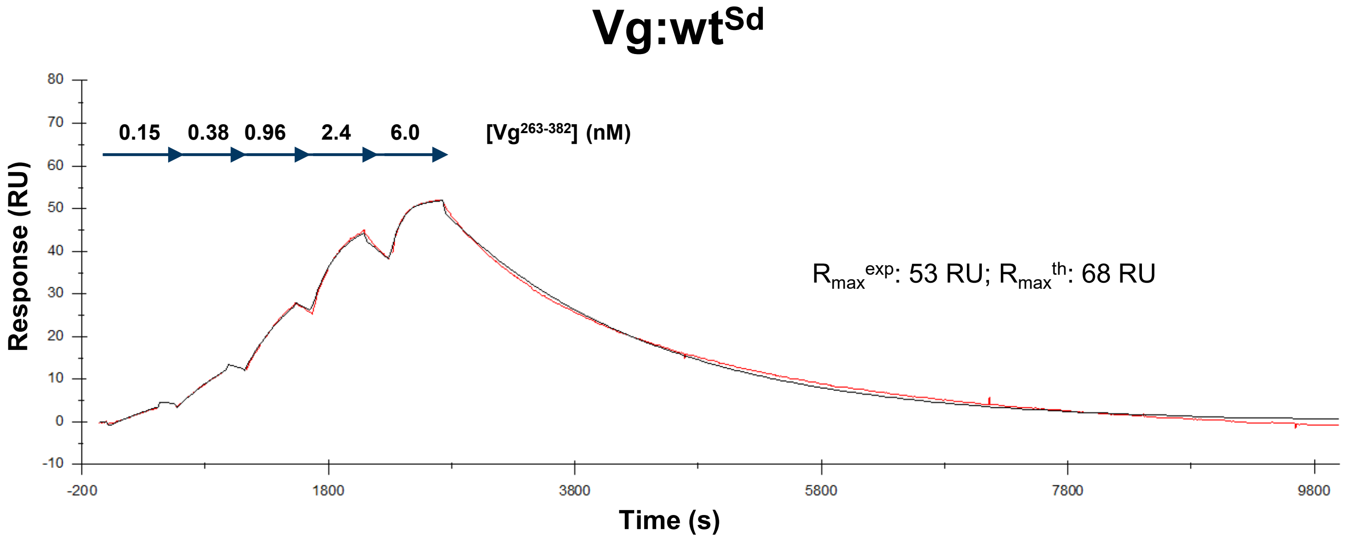

Vg:wtSd
0.15
0.38
0.96
2.4
6.0
[Vg263-382] (nM)
Rmax exp: 53 RU; Rmax th: 68 RU
Response (RU)
Time (s)

Figure S4

C

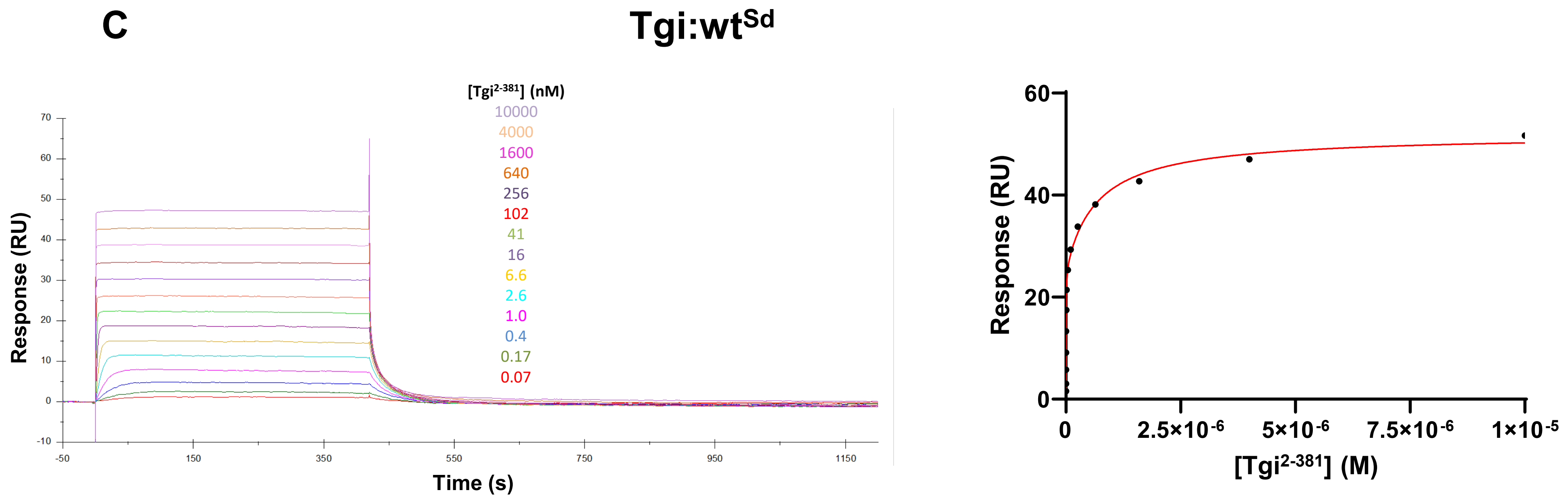
Tgi:wt^Sd
[Tgi2-381] (nM)
10000
4000
1600
640
256
102
41
16
6.6
2.6
1.0
0.4
0.17
0.07
Response (RU)
Time (s)
[Tgi2-381] (M)

D

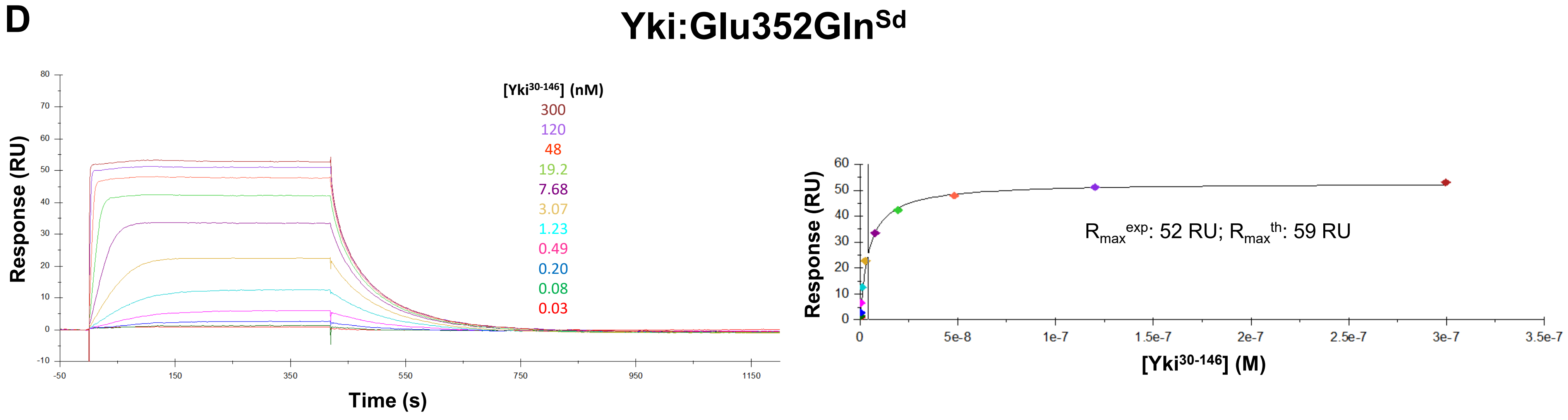
Yki:Glu352Gln^Sd
[Yki30-146] (nM)
300
120
48
19.2
7.68
3.07
1.23
0.49
0.20
0.08
0.03
Response (RU)
Time (s)
Rmax exp: 52 RU; Rmax th: 59 RU
[Yki30-146] (M)

Figure S4

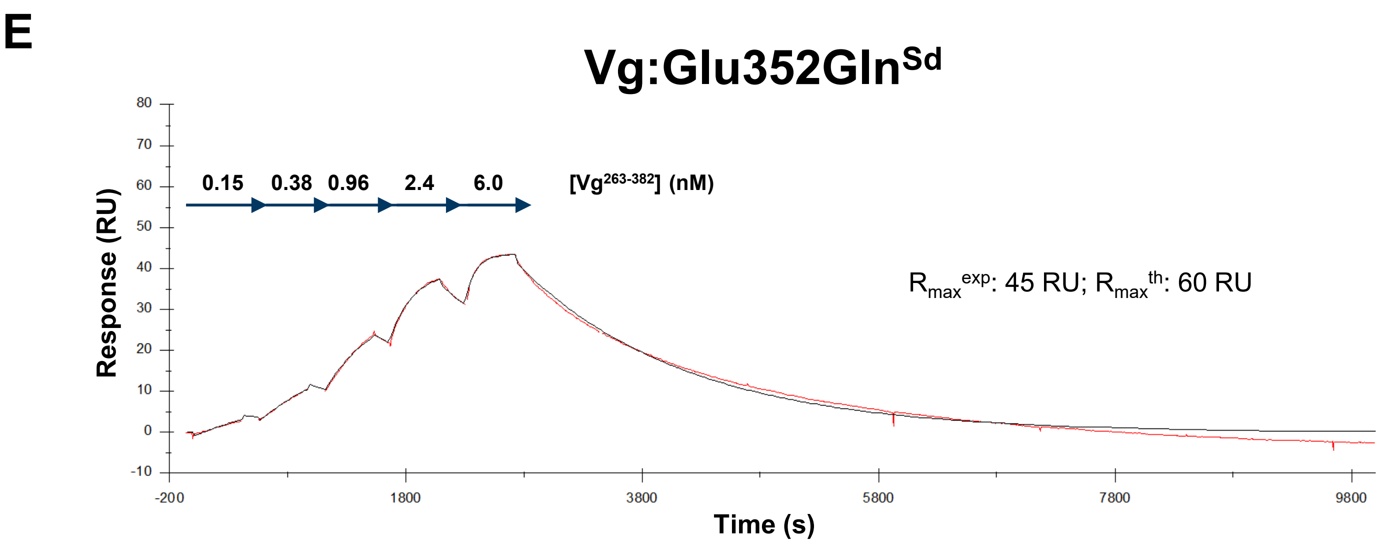
E
Vg:Glu352Gln^{Sd}
0.15
0.38
0.96
2.4
6.0
[Vg$^{263-382}$] (nM)
R_{max}^{exp}: 45 RU; R_{max}^{th}: 60 RU
Response (RU)
Time (s)

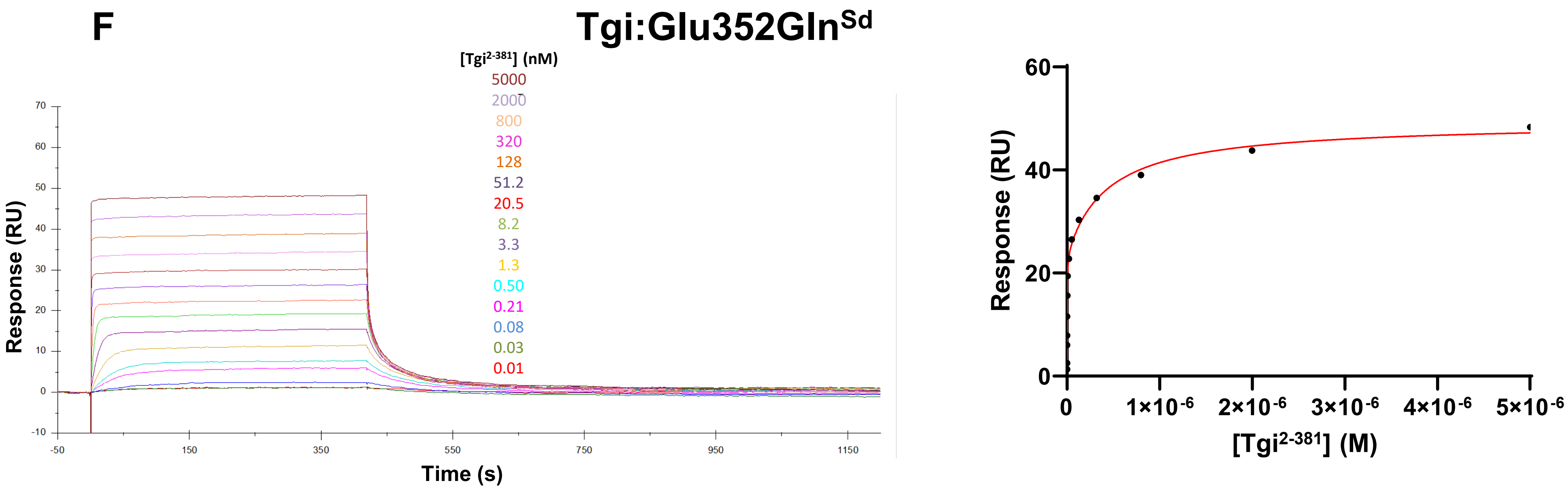
F
Tgi:Glu352Gln^{Sd}
[Tgi$^{2-381}$] (nM)
5000
2000
800
320
128
51.2
20.5
8.2
3.3
1.3
0.50
0.21
0.08
0.03
0.01
Response (RU)
Time (s)
[Tgi$^{2-381}$] (M)

Figure S4

G

Yki:Lys350Ala^{Sd}

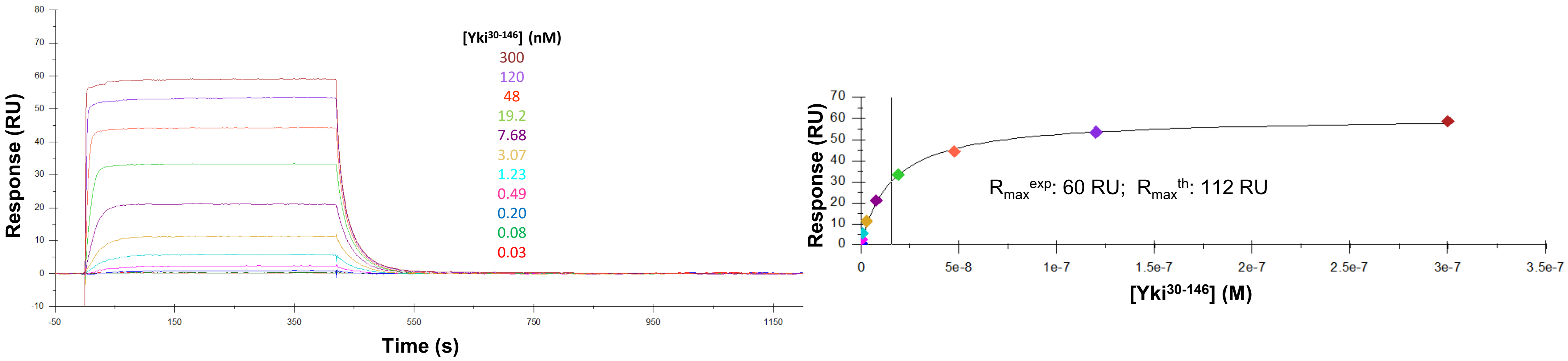

Vg:Lys350Ala^{Sd}

H

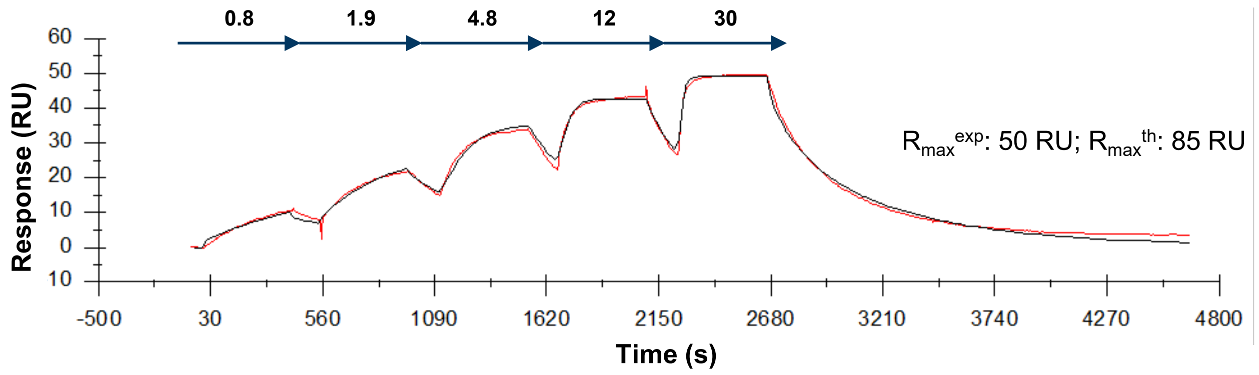

Figure S4

I

Tgi:Lys350Ala^{Sd}

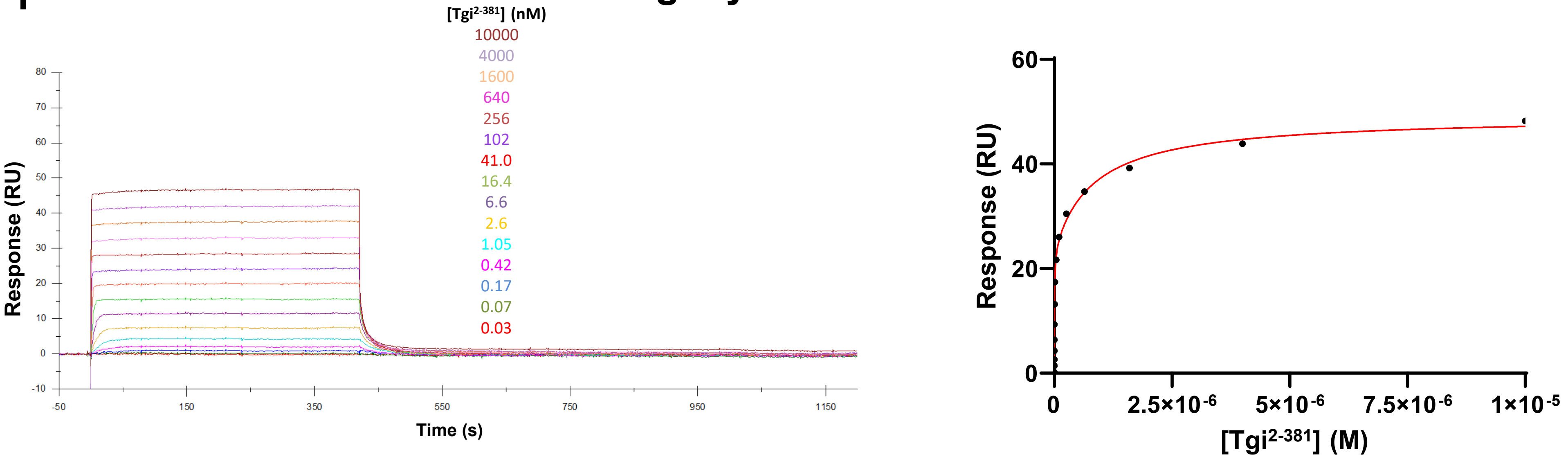

Figure S5

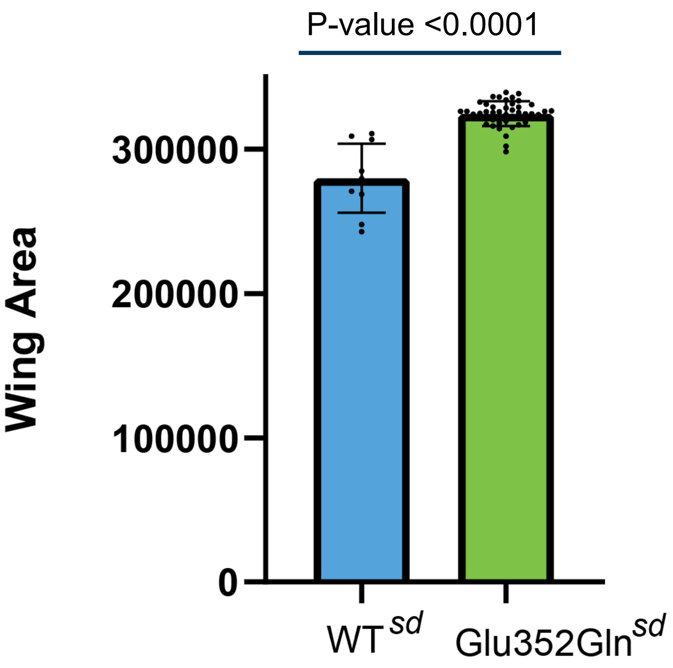

P-value <0.0001
Wing Area
300000
200000
100000
0
WT sd
Glu352Gln sd

Figure S6

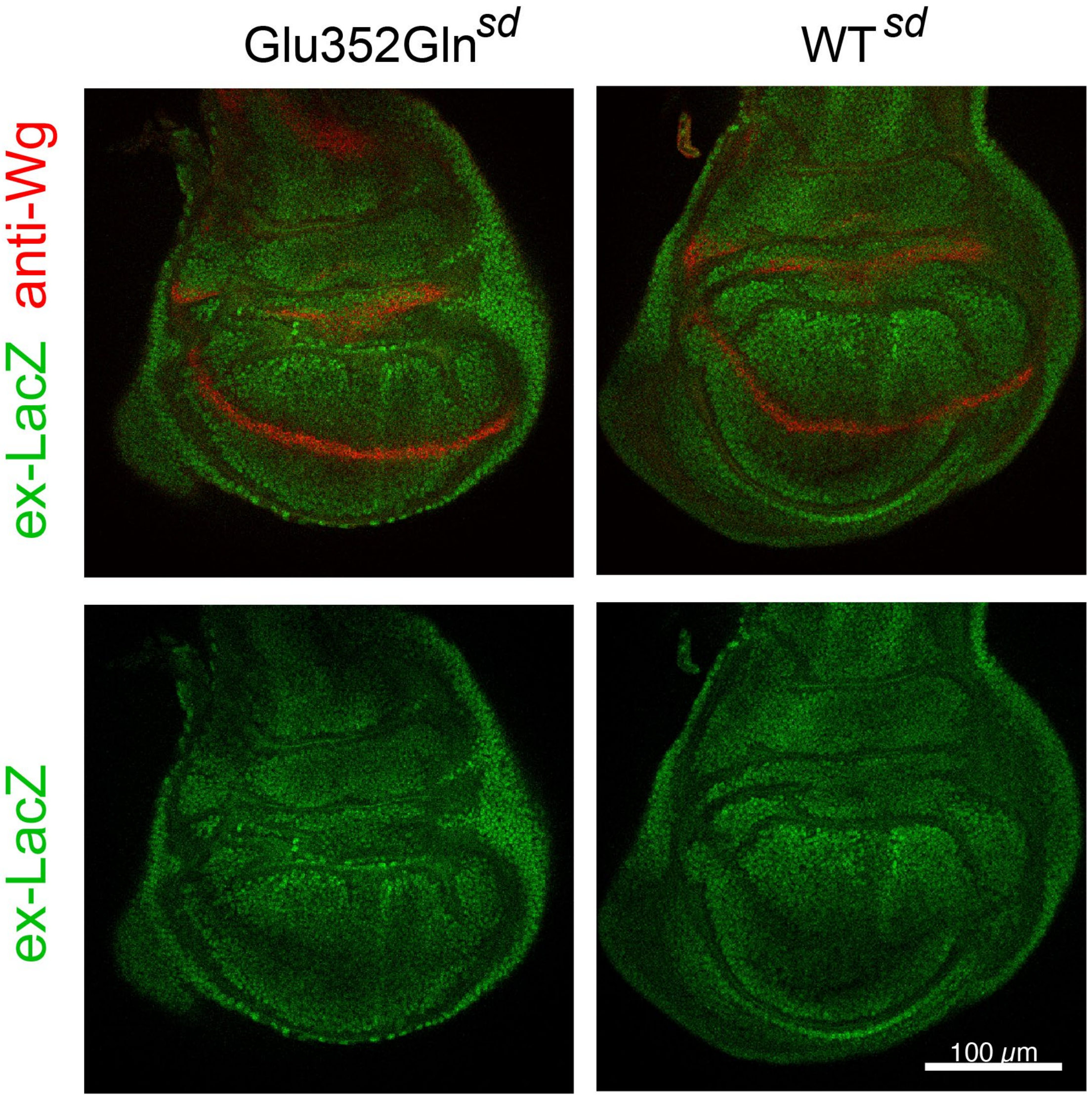

Supplement: Supplementary file 1 — Supplementary Information. [file 41598_2022_9127_MOESM1_ESM.pdf]
